# Supplementary material for: Role for gene conversion in the evolution of cell-surface antigens of the malaria parasite Plasmodium falciparum
Source: PLoS Biol. 2024 Mar 7;22(3):e3002507. doi: 10.1371/journal.pbio.3002507 (PMC10919680; doi:10.1371/journal.pbio.3002507)
Supplement: S1 Text — (DOCX) [file pbio.3002507.s001.docx]

### Supplementary text for ‘Role for gene conversion in the evolution of cell-surface antigens of the malaria parasite *Plasmodium falciparum*’

#### New genotyping pipeline

In S4 Fig, we illustrate MalariaGEN’s existing pipeline (panel a) and our new pipeline (panel b). Both approaches first genotype samples individually and then re-genotype each sample at the union of all variants found (joint genotyping). MalariaGEN uses GATK for both steps (1), while in our new pipeline per-sample calls are obtained using a range of tools (Cortex (2), Octopus (3), gramtools (4) and Gapfiller (5)) and joint genotyping is performed using our genome-graph-based genotyper, gramtools. Compared to our previous work (4,6), we added the tool Octopus (3) prior to adjudication and GapFiller (5) after adjudication, to resolve remaining diverged alleles.

In S1 Table, we outline the methodology and strengths of each step of our new pipeline. Cortex can assemble large diverged alleles and is highly specific (low false-positive rate), but misses many alleles (low sensitivity). To recover sensitivity, we use Octopus, a pileup-based variant caller (like GATK), but that outperformed GATK in their benchmark, and pays attention to indel-calling in repeat-rich regions (3). We then combine the output of both Octopus and Cortex using our genome-graph-based genotyping tool, gramtools, in a process we call *adjudication* and introduced in (6). The essential idea is to map reads to a graph containing alternate variant calls, and use the mapping of the reads to determine which caller (here, Cortex or Octopus) was correct, when they conflict. After adjudication, we run Gapfiller, a semi-global assembly-based caller, to recover remaining missing diverged alleles. Gapfiller can efficiently assemble the sequence between aligned read pairs using both reads mapped in the region and all unmapped reads (5).

Finally, we perform joint genotyping with gramtools. We built a genome graph containing the variation found in all analysed samples across both genes (plus the 3D7 reference sequence for the rest of the genome), and genotyped each sample using this graph. This enables recovering variants missed in an individual sample, but present in the graph by having been

recovered in other samples.

#### Genotyping evaluation framework and performance

All evaluations started from VCF files of variant calls (7).

We used two different approaches to evaluate variant calls, both illustrated in S5 Fig. The first approach used 14 samples sequenced and assembled by Otto et al. (2018) (8) using both Illumina (MiSeq, 250bp paired-end) and PacBio technologies. We genotyped the samples using the Illumina data only and compared the output to the assemblies, considering them as the true sample genomes. At each step of our pipeline, variant calls made were applied to the 3D7 reference gene sequence and the resulting ‘induced gene sequence’ was mapped to each sample's truth assembly. Performance was measured as the edit distance between the two sequences divided by the gene length (S5 Fig panel a). A value of zero indicates perfect agreement between the tool's calls and the truth, while values >0 indicate missing and/or false positive calls. Note that the 14 samples were not part of the 3,589 analysed samples, and thus did not figure in the genome graph used for the joint genotyping step.

To evaluate genotyping calls in the 3,589 analysed samples themselves - i.e., in the absence of a source of independent truth - we used a read realignment-based approach. At each pipeline step, called variants were applied to the *P. falciparum* reference genome to create an ‘induced reference genome’ and the reads remapped to it. Missing or incorrect variants can be identified by looking for inconsistencies between the aligned reads pileups and the induced reference (S5 Fig panel b). Specifically, we measured for each gene the number of positions where the majority of reads disagree with the reference base, the number of positions with no (or low) read coverage, and the number of read pairs with abnormal insert sizes (i.e. falling outside the bulk of the size distribution).

In S6 Fig, we show the evaluation results for each step of the gramtools-based pipeline in both DBLMSP and DBLSMP2. Panels a and b correspond to those in S5 Fig. Across both panels, and for both genes, each step of the genotyping pipeline improves (or is at least as good as) the previous one, and all metrics approach zero after the final step (‘gram_joint_geno’, beige colour). In panel b, only a subset of 500 samples was evaluated, selected to be as geographically wide-ranging as possible, to save on compute and storage requirements (406GB of disk for 500 samples compared to 2.8TB for all samples). For comparing our pipeline and the MalariaGEN results (see below), all samples were evaluated.

In S7 Fig, we compare the results of the final step of the gramtools-based pipeline with the final output of the GATK-based pipeline, showing the same metrics as in S6 Fig. For GATK, results on the 3,589 analysed samples (panel b) were taken from the 2021 release (9), while for the 14 truth assemblies (panel a) they were taken from the 2023 release (10). For both genes, our pipeline clearly outperforms the GATK-based approach on all metrics.

In S6 Fig and S7 Fig, only the mean values across samples are shown. We show the full frequency distribution for the data in S8 Fig.

Based on the realignment metrics computed on the 3,589 analysed samples (S7 Fig panel b), we designed a set of criteria to produce a final set of DBLMSP1/2 sequences for analysis. To consider a sequence for downstream analysis, we required three conditions to hold: i) none of the gene positions contained fewer than five aligned reads (pileup-based gaps); ii) none of the gene positions contained pileup-based differences, defined as a position where the majority of aligned bases differed from the induced reference; iii) the fraction of reads with large insert sizes was low, where an insert size was defined as large if it exceeded the mean insert size + two standard deviations. We defined ‘large fraction’ as 15% by looking at the distribution of the fraction of large insert sizes across the 3,589 analysis samples in a gene that is confidently-resolved by both pipelines (AMA1), and taking the value below which almost all samples lie.

The first two filters ensured that the remapped reads fully covered and agreed with the inferred gene sequence, and also removed samples with low-coverage, due for example to DNA amplification bias. The last filter ensured that large structural variations were not missed: for example, translocations or duplications can lead to abnormally large insert sizes. S9 Fig shows the number of sequences left after each filter is successively applied, across both DBLMSP and DBLMSP2 (x-axis). The three vertical panels show a ‘baseline’ approach of making no calls, and the two genotyping pipelines (‘gram_joint_geno’, ‘malariaGEN’).

In total, 5,895 DBLMSP1/2 sequences from our pipeline met all three criteria (5,893 from S9 Fig (2,929 DBLMSP and 2,964 DBLMSP2 sequences), plus the two 3D7 reference sequences). For an additional 200 DBLMSP1/2 sequences, criteria i) and iii) were met, but a single pileup-based difference remained in the remapped reads, with a clear single base differing from the induced reference at a frequency >0.5 in the reads. We corrected these single SNPs with a custom script (available with this paper, see data availability) and added these sequences to our analysis set. We also added the 28 DBLMSP1/2 sequences from the 14 samples with truth assemblies by Otto et al. (8), giving a total of 6,123 analysed sequences.

#### Sequence sharing patterns

*Shared and private peptides*

To define sequence sharing, we broke each sequence in our multiple-sequence alignment (MSA) of all confidently-resolved DBLMSP1/2 sequences into overlapping peptides of length 10 (*10-mers*), and called a 10-mer *shared* if it was seen at least once in each gene at a given position in the MSA and *private* if not. This definition does not consider geography, meaning, for example, a peptide could be called shared but be seen only once on DBLMSP in a sample from Ghana and once in DBLMSP2 in Cambodia. An alternative is to require a

peptide to be found in the same geographical unit (e.g. country) at least once to be

considered shared, making them more likely to have a common ancestor and be

truly related (as opposed to, for example, convergent mutations). We found

both definitions to be essentially equivalent, as most shared peptides by our definition

are also found on both genes in at least one country (S10 Fig).

We illustrate shared peptides in S11 Fig: the two innermost rings are the same as Fig 2 of the main text, and the outermost ring colours each sequence by the fraction of shared 10-mer peptides it possesses. While lineage A and C are mostly ‘private’ (low shared fraction), all lineage B members have a high fraction of shared kmers (>0.5, green colour).

*HMM logos*

To produce HMM logos of the private and shared lineages, we first split the original MSA into three separate MSAs, assigning peptides in the original sequences based on private or shared status. For each gene sequence in each sample, each peptide 10-mer was pasted to either the gene’s private MSA, or the shared MSA, adding gaps (‘-’ character) where necessary (the sample’s entry in the shared MSA receives gaps for a 10-mer assigned to the private MSA, and vice-versa). We then built one HMM model per MSA using hmmer, and produced logos for visualisation using Skylign (11). In the HMM logos, each letter’s relative height is proportional to its relative frequency in the MSA, and the total height of each stack of letters is proportional to ‘information content’, a measure of how different the observed amino acid distribution is from a background expectation (e.g. based on Swiss-prot; though total height is not particularly important here).

The resulting HMM logos are shown in S16 Fig. Tracks labelled ‘DBLMSP’ and ‘DBLMSP2’ show the private peptides, and ‘Both’ show the shared peptides. Either side of the DSR only private peptides are found, meaning the genes have diverged (as also visible in Fig 1 of the main text). Inside the DSR, two amino acids are commonly found in each gene - one private and one shared - interspersed with fixed amino acids (e.g. the well-known ‘PPRR’ motif of DBL domains, and a number of cysteine and tryptophan residues) and some more highly polymorphic locations (e.g. the C-terminal region of the DSR). Some low-frequency amino acids additionally exist, though not visible in S16 Fig (see S17 Fig).

## References

1. Poplin R, Ruano-Rubio V, DePristo MA, Fennell TJ, Carneiro MO, Auwera GAV der, et al. Scaling accurate genetic variant discovery to tens of thousands of samples. bioRxiv. 2018 Jul 24;201178.

2. Iqbal Z, Caccamo M, Turner I, Flicek P, McVean G. *De novo* assembly and genotyping of variants using colored de Bruijn graphs. Nat Genet. 2012 Feb;44(2):226–32.

3. Cooke DP, Wedge DC, Lunter G. A unified haplotype-based method for accurate and comprehensive variant calling. Nat Biotechnol. 2021 Jul;39(7):885–92.

4. Letcher B, Hunt M, Iqbal Z. Gramtools enables multiscale variation analysis with genome graphs. Genome Biol. 2021 Dec;22(1):259.

5. Nadalin F, Vezzi F, Policriti A. GapFiller: a de novo assembly approach to fill the gap within paired reads. BMC Bioinformatics. 2012 Sep 7;13(14):S8.

6. Hunt M, Letcher B, Malone KM, Nguyen G, Hall MB, Colquhoun RM, et al. Minos: variant adjudication and joint genotyping of cohorts of bacterial genomes. Genome Biol. 2022 Jul 5;23(1):147.

7. Danecek P, Auton A, Abecasis G, Albers CA, Banks E, DePristo MA, et al. The variant call format and VCFtools. Bioinformatics. 2011 Aug 1;27(15):2156–8.

8. Otto TD, Böhme U, Sanders M, Reid A, Bruske EI, Duffy CW, et al. Long read assemblies of geographically dispersed Plasmodium falciparum isolates reveal highly structured subtelomeres. Wellcome Open Res. 2018 May 3;3.

9. MalariaGEN, Ahouidi A, Ali M, Almagro-Garcia J, Amambua-Ngwa A, Amaratunga C, et al. An open dataset of Plasmodium falciparum genome variation in 7,000 worldwide samples. Wellcome Open Res. 2021 Feb 24;6:42.

10. MalariaGEN, Abdel Hamid MM, Abdelraheem MH, Acheampong DO, Ahouidi A, Ali M, et al. Pf7: an open dataset of Plasmodium falciparum genome variation in 20,000 worldwide samples. Wellcome Open Res. 2023 Jan 16;8:22.

11. Wheeler TJ, Clements J, Finn RD. Skylign: a tool for creating informative, interactive logos representing sequence alignments and profile hidden Markov models. BMC Bioinformatics. 2014 Dec;15(1):7.
