# Supplementary figures and images for: Role for gene conversion in the evolution of cell-surface antigens of the malaria parasite Plasmodium falciparum
Source: PLoS Biol. 2024 Mar 7;22(3):e3002507. doi: 10.1371/journal.pbio.3002507 (PMC10919680; doi:10.1371/journal.pbio.3002507)

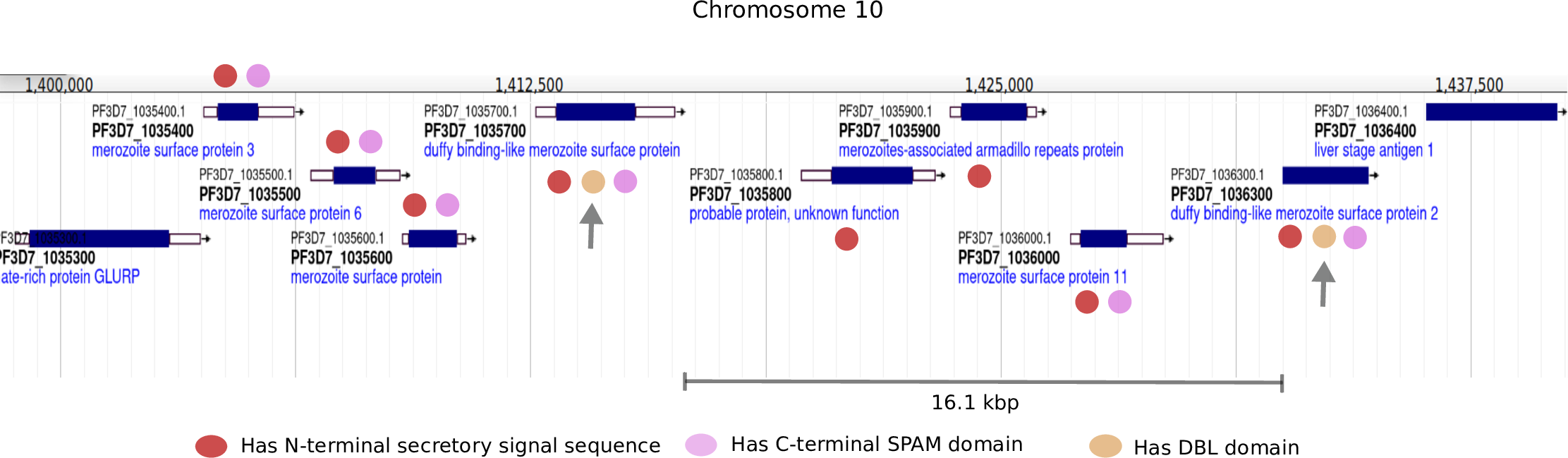

Supplement: S1 Fig — The 2 genes, marked with grey arrows, are located at a distance of 16.1 kbp from each other, inside an array of 8 contiguous genes spanning 32 kbp on chromosome 10. These genes are likely paralogs due to observed sequence sharing: All 8 have an N-terminal shared motif, a further 6 have a C-terminal SPAM domain, and DBLMSP and DBLMSP2 further share a DBL domain (domains shown as coloured circles below each gene). Figure annotated from a screenshot of the gene track for DBLMSP2 taken from PlasmoDB. (TIF) [file pbio.3002507.s002.tif]

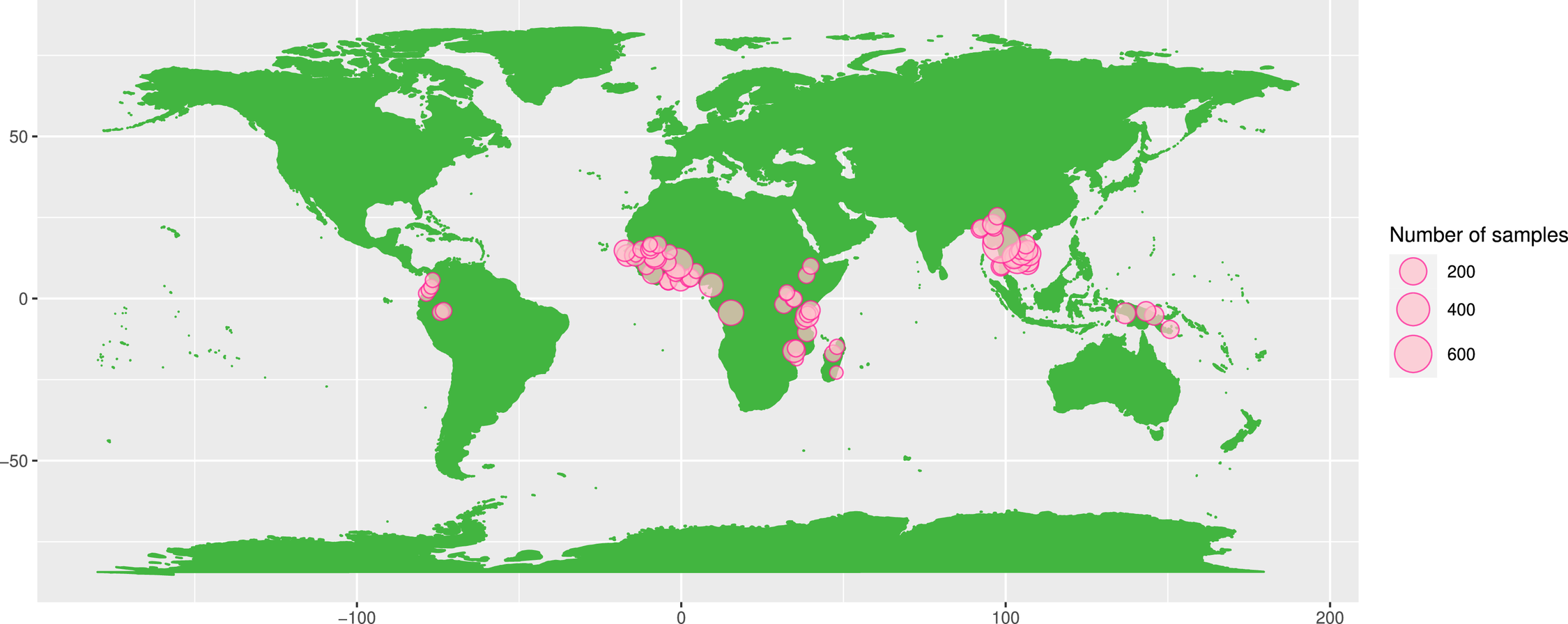

Supplement: S2 Fig — A total of 29 countries are represented, with most samples located in the 2 regions with highest endemicity: sub-Saharan Africa and Southeast Asia. The base map comes from the freely distributed R package “maps,” under a GPL-2 licence: https://cran.r-project.org/package=maps. The data and code to generate this Figure can be found at https://zenodo.org/doi/10.5281/zenodo.7677547. (TIF) [file pbio.3002507.s003.tif]

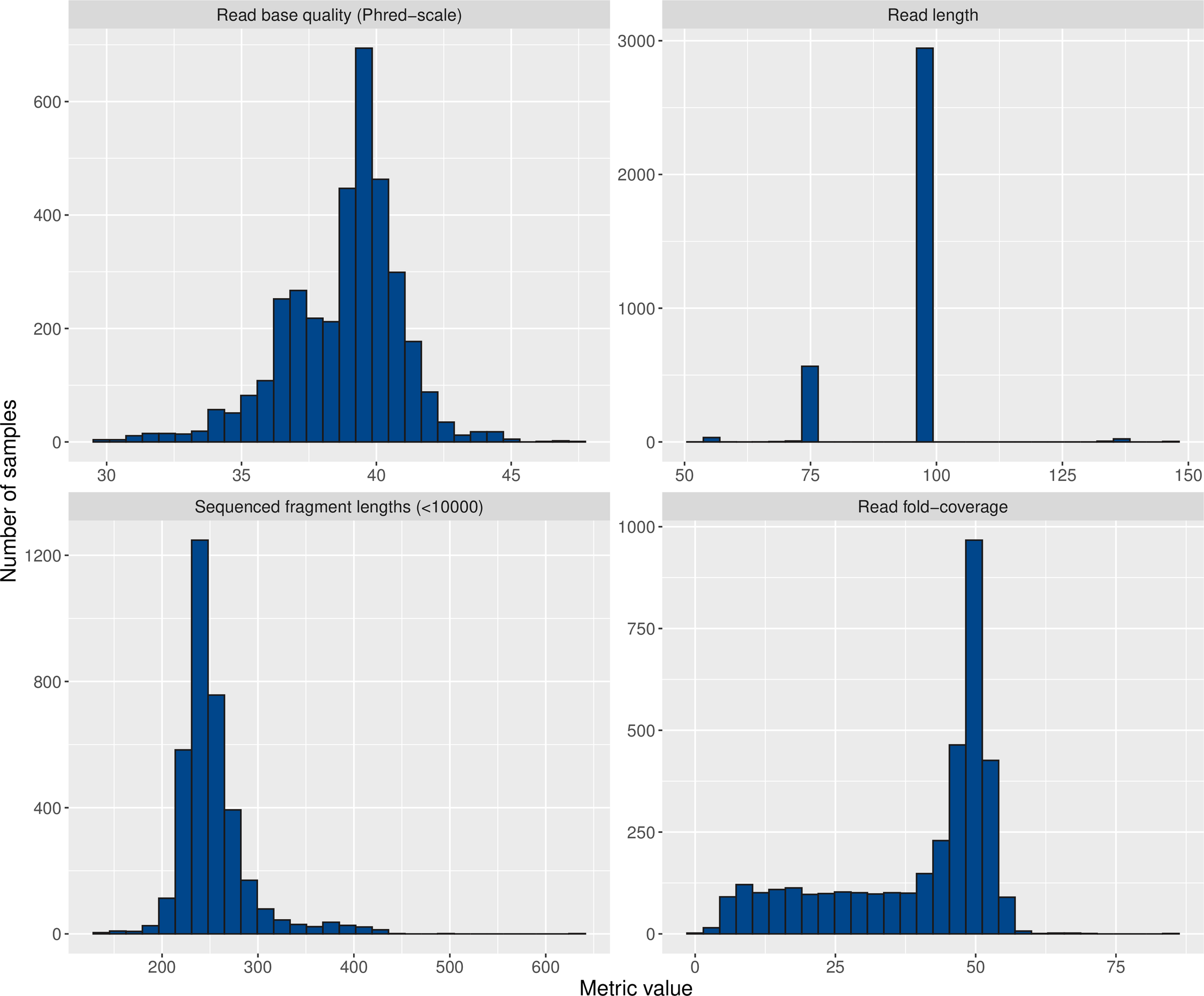

Supplement: S3 Fig — The upper panels show statistics measured on the reads directly: per-base quality (top-left panel) and read lengths (top-right panel). Per-base quality q gives the Illumina-estimated sequencing error rate ϵ as ϵ=10−q10. The lower panels show statistics measured after mapping the reads to the P. falciparum 3D7 reference genome: fragment length, estimated from the distance between paired-end reads (bottom-left) and fold-coverage, estimated from the number of reads at each mapped position. The data and code to generate this Figure can be found at https://zenodo.org/doi/10.5281/zenodo.7677547. (TIF) [file pbio.3002507.s004.tif]

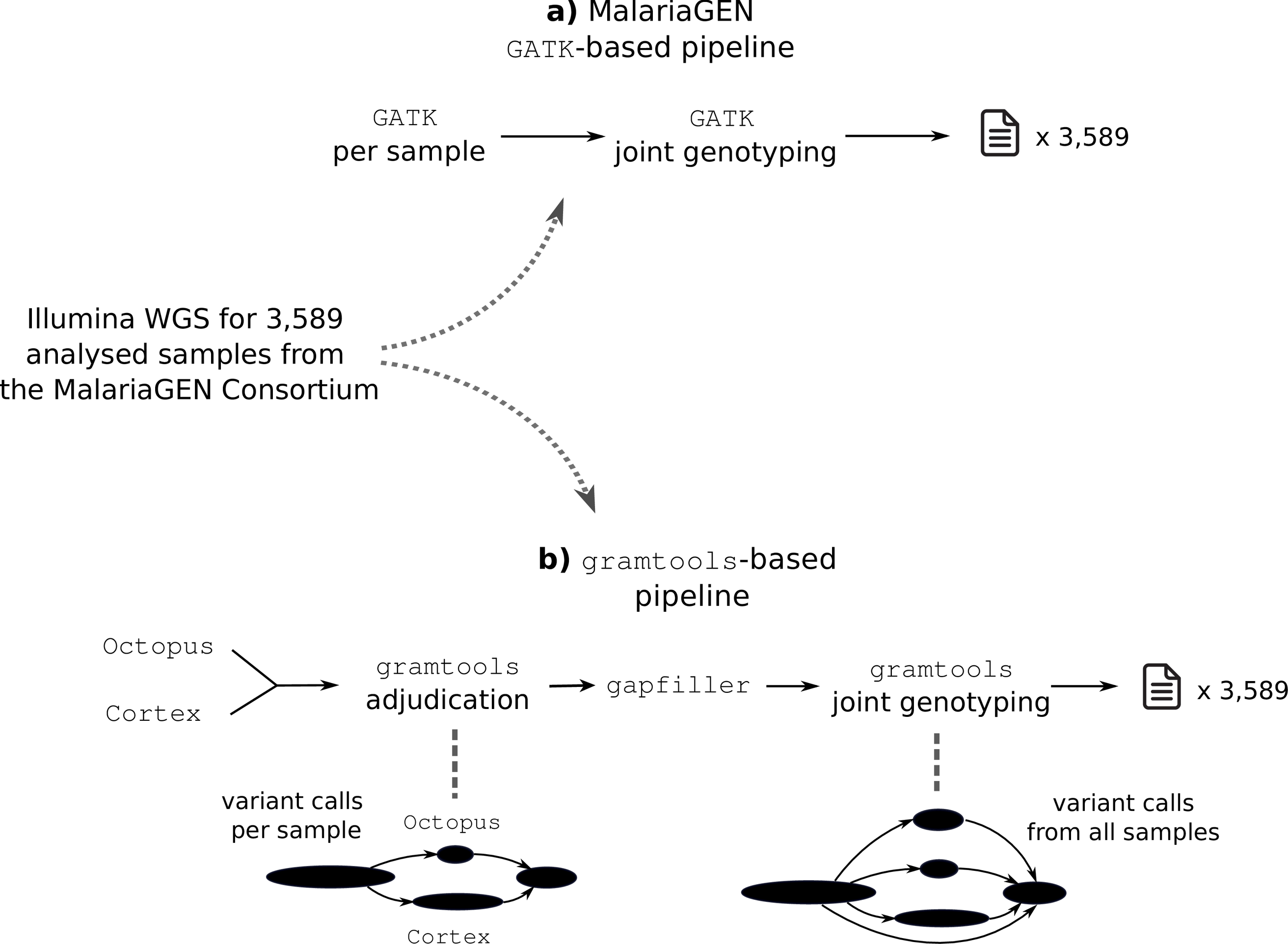

Supplement: S4 Fig — Panel (a) illustrates MalariaGEN’s existing GATK-based pipeline, and panel (b) illustrates our new pipeline. Both first discover variants in each sample individually before regenotyping each sample at the union of all variants. GATK relies on the linear reference genome to do this, while gramtools uses a genome graph. (TIF) [file pbio.3002507.s005.tif]

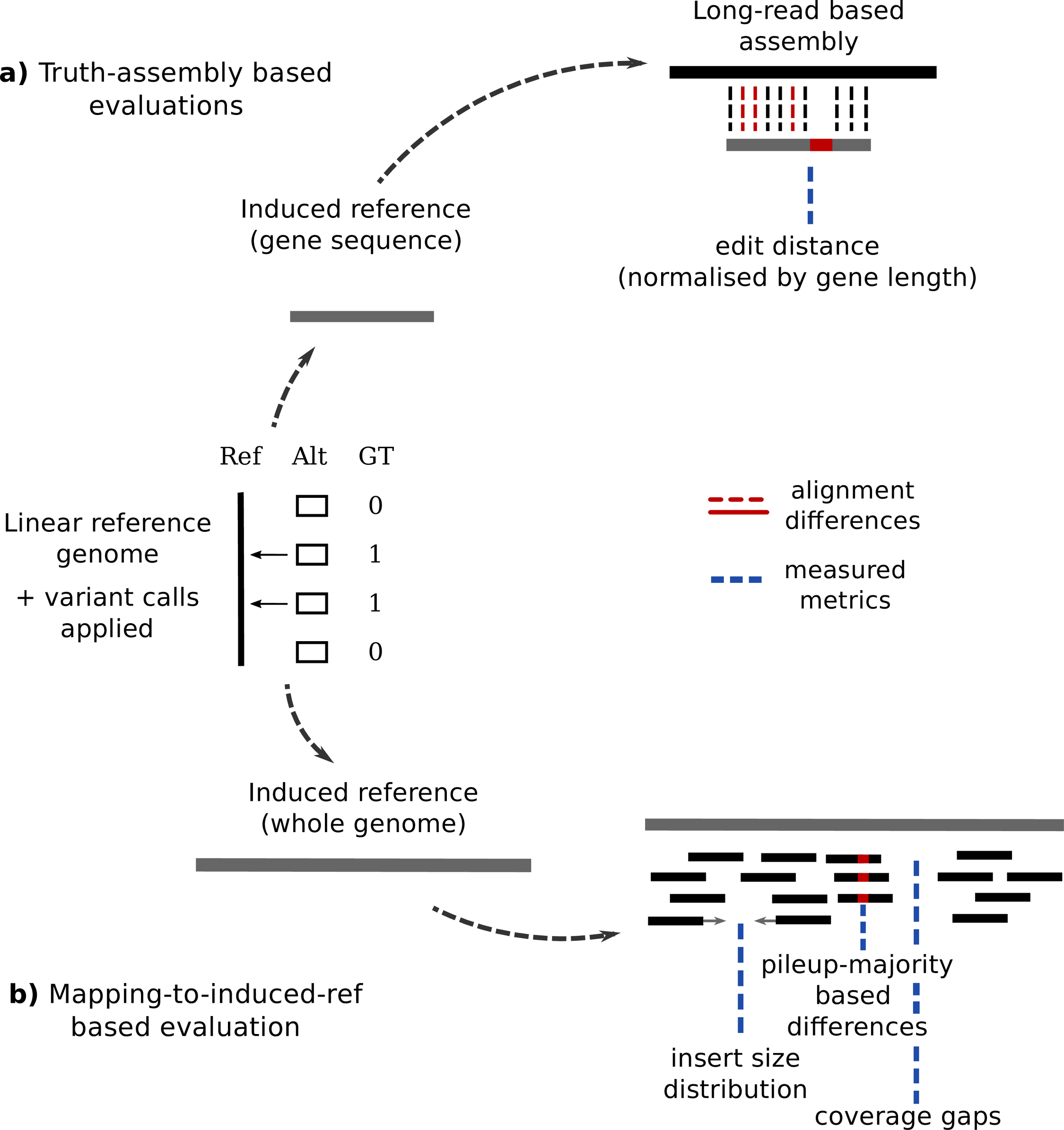

Supplement: S5 Fig — Starting from a tool’s variant calls in a VCF file (middle), 2 independent evaluations were performed. First, for 14 samples with truth assemblies, the calls were directly compared to the truth, by applying them to the 3D7 gene sequence and measuring edit distance for the whole gene (part a). Second, the calls were all applied to the reference genome, and the reads remapped to this induced reference. Incorrect or missing calls then appear from read pileups, as majority-differences compared to the reference base, coverage gaps, or inconsistent insert sizes between read pairs (part b). (TIF) [file pbio.3002507.s006.tif]

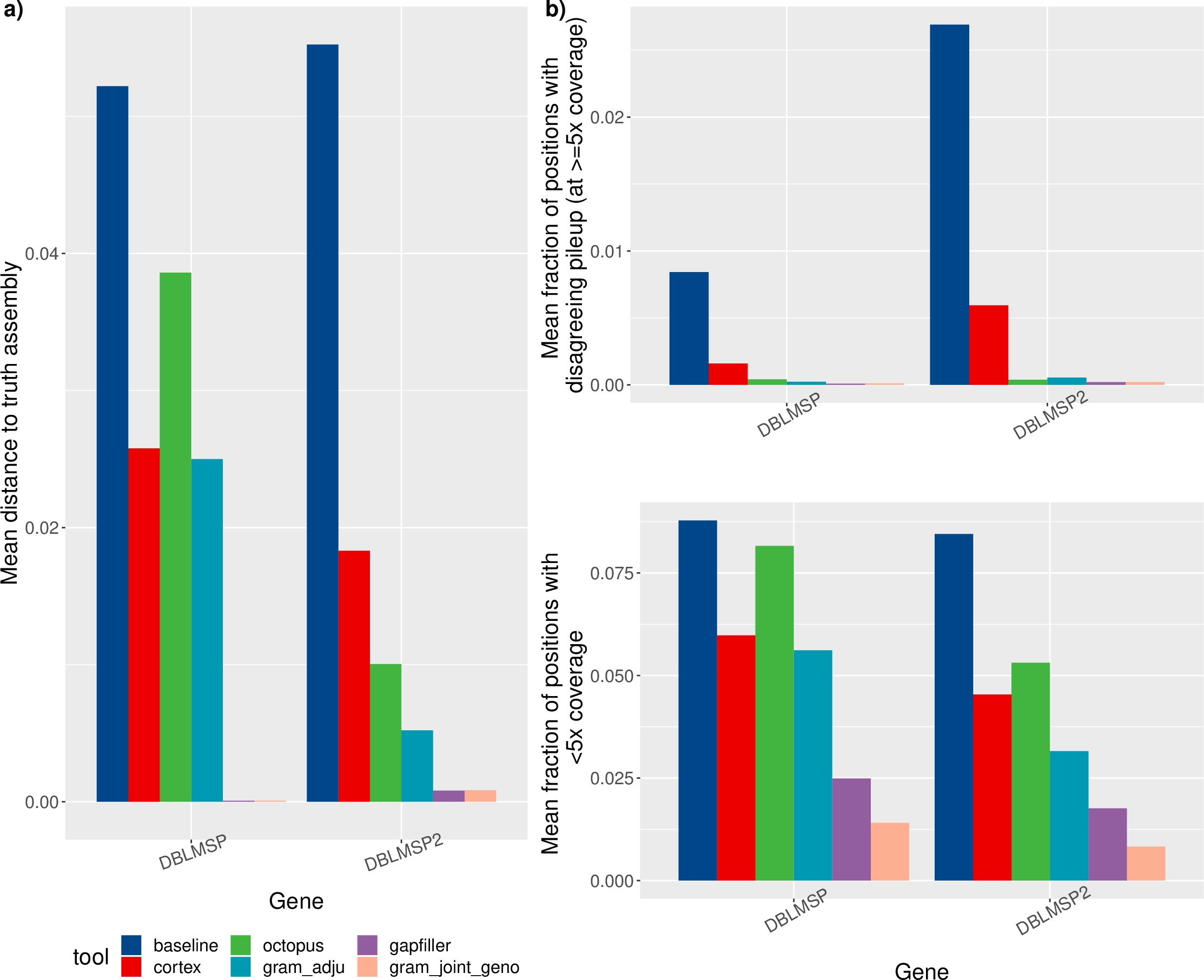

Supplement: S6 Fig — The 2 panels, a and b, correspond to parts a and b of the evaluation framework in S5 Fig. Panel (a) shows the mean edit distance between the inferred gene sequence and the truth assembly for the 14 samples with truth assemblies (edit distance is scaled by gene length). Panel (b) shows the fraction of positions with pileup-based differences (top) and with low read coverage (bottom), after the sequencing reads are remapped to the 3D7 reference genome with each tool’s called variants applied. A pileup-based difference is when the majority of reads disagree with the reference at a given position, given a minimum of 5 mapped reads, and low read coverage is defined as a position with fewer than 5 mapped reads. Each bar in panel b shows the mean across 500 of the 3,589 analysed samples. Across both panels, each coloured bar corresponds to one additional step in the gramtools-based pipeline, in the same order they are run (see S4 Fig). The “baseline” condition is not part of the pipeline and refers to using 3D7 reference gene sequence with no variants applied (in panel a: 3D7 sequence aligned to the truth assemblies; in panel b: sample reads aligned to the 3D7 reference genome). The data and code to generate this Figure can be found at https://zenodo.org/doi/10.5281/zenodo.7677547. (TIF) [file pbio.3002507.s007.tif]

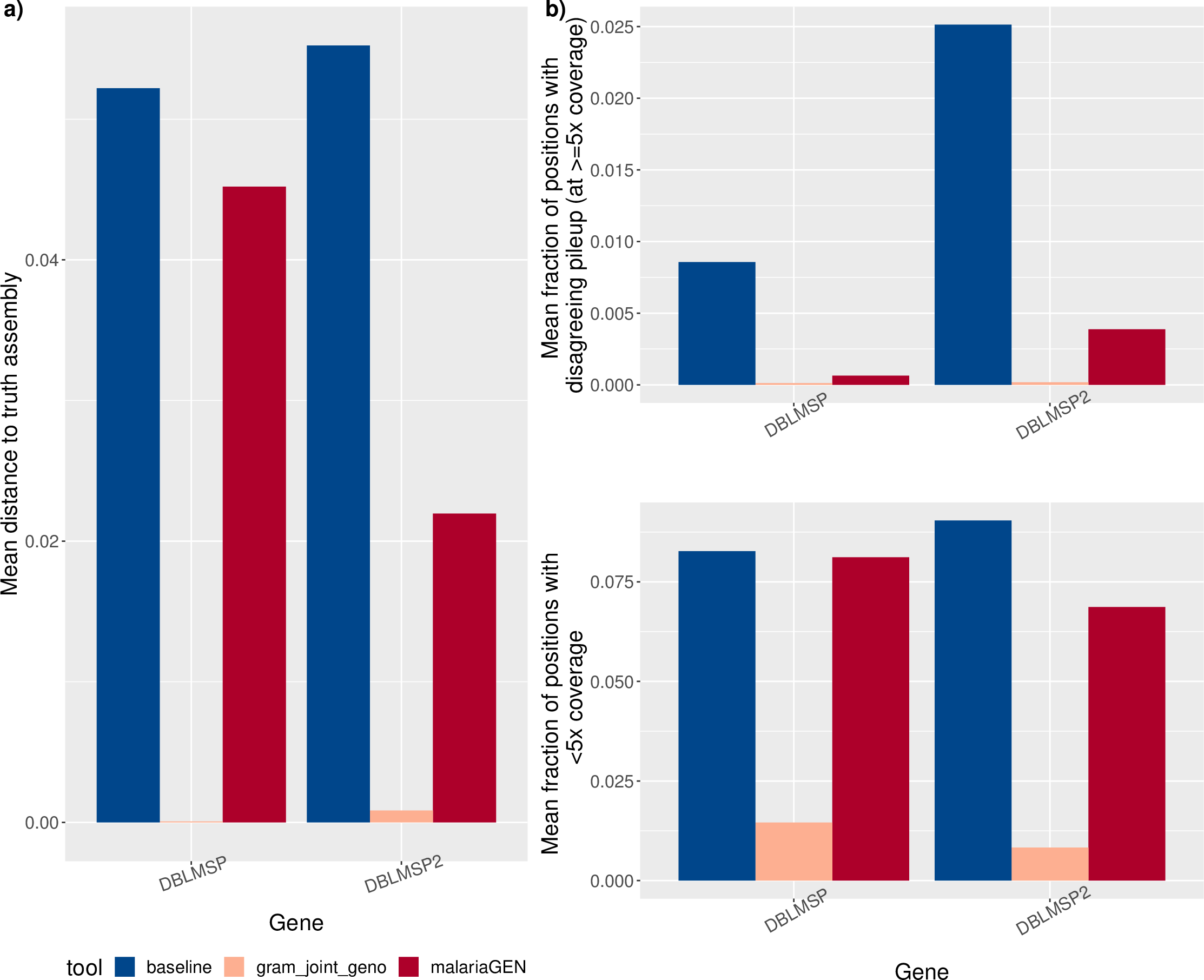

Supplement: S7 Fig — Panels a and b show the same metrics as S6 Fig. Metrics in panel b were computed on all 3,589 analysed samples. The data and code to generate this Figure can be found at https://zenodo.org/doi/10.5281/zenodo.7677547. (TIF) [file pbio.3002507.s008.tif]

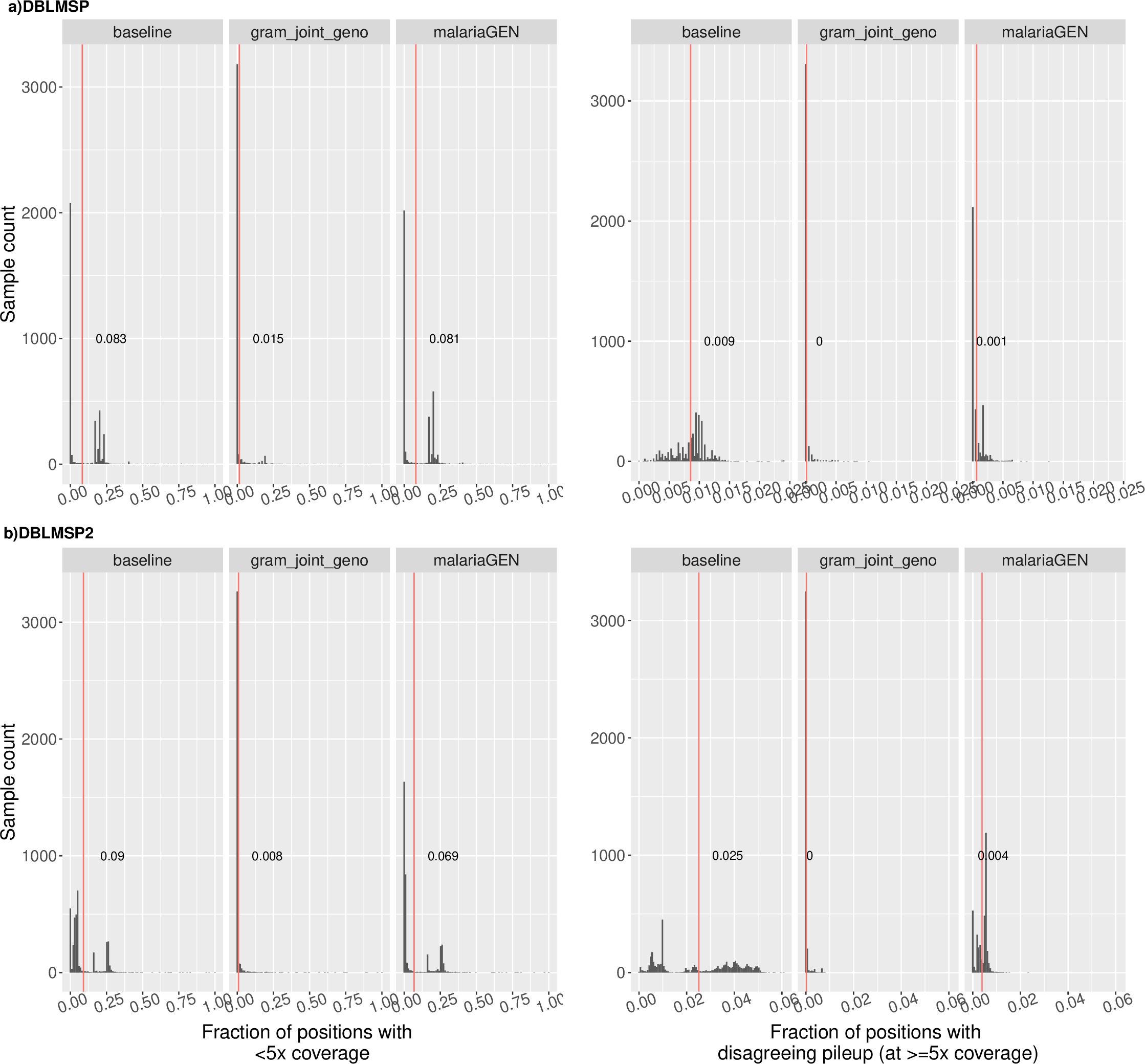

Supplement: S8 Fig — Each subplot shows the frequency distribution, across all 3,589 analysed samples, of the fraction of positions with pileup-based gaps (left-hand side plots) or differences (right-hand side plots), for DBLMSP (top) and DBLMSP2 (bottom). The mean is shown as a red vertical line (value shown in text next to it) and corresponds to the height of the coloured bars in S7 Fig panel b. The data and code to generate this Figure can be found at https://zenodo.org/doi/10.5281/zenodo.7677547. (TIF) [file pbio.3002507.s009.tif]

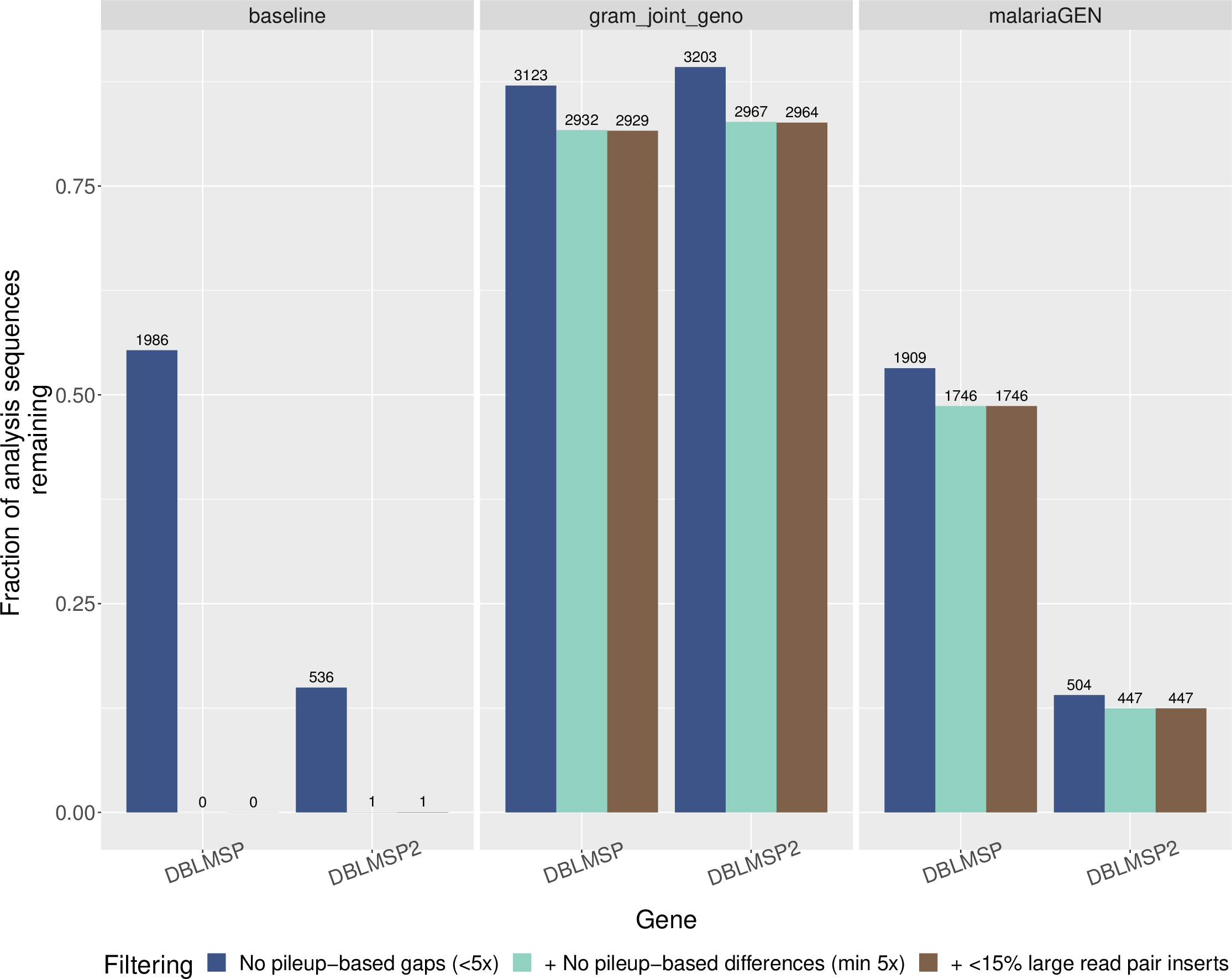

Supplement: S9 Fig — In each panel (“baseline”:no variant calling, “gram_joint_geno”: gramtools-based pipeline, “malariaGEN”: GATK-based pipeline), the total fraction of remaining gene sequences (out of the 3,589 analysed samples) passing filters is shown. Filters (colours) are applied in succession, on each set of remaining gene sequences, in the order they appear in the legend. The number of remaining sequences is given above each coloured bar. The data and code to generate this Figure can be found at https://zenodo.org/doi/10.5281/zenodo.7677547. (TIF) [file pbio.3002507.s010.tif]

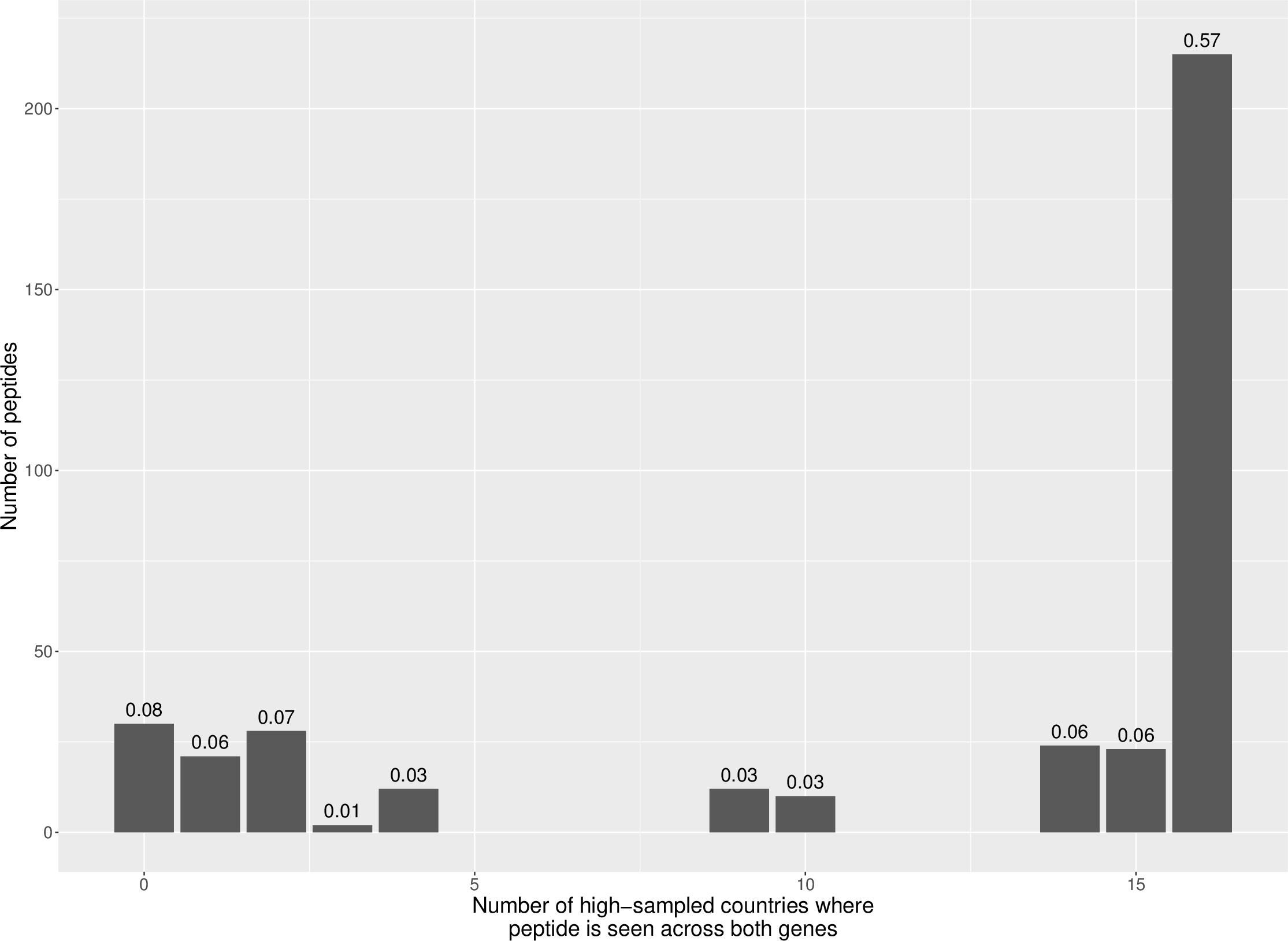

Supplement: S10 Fig — The number of shared peptides by our definition (y-axis) that are found in both genes inside the same country, for up to 16 countries with high levels of sampling (defined as >50 available DBLMSP1/2 sequences; x-axis). A value of zero on the x-axis means the shared peptide is not found on both genes in any of these countries, and 16 means it is found on both genes in all of them. A majority (57%) of shared peptides are found in all of these countries, and 86% are found in at least 2 different countries, showing that the shared peptides are, overall, highly widespread geographically. The data and code to generate this Figure can be found at https://zenodo.org/doi/10.5281/zenodo.7677547. (TIF) [file pbio.3002507.s011.tif]

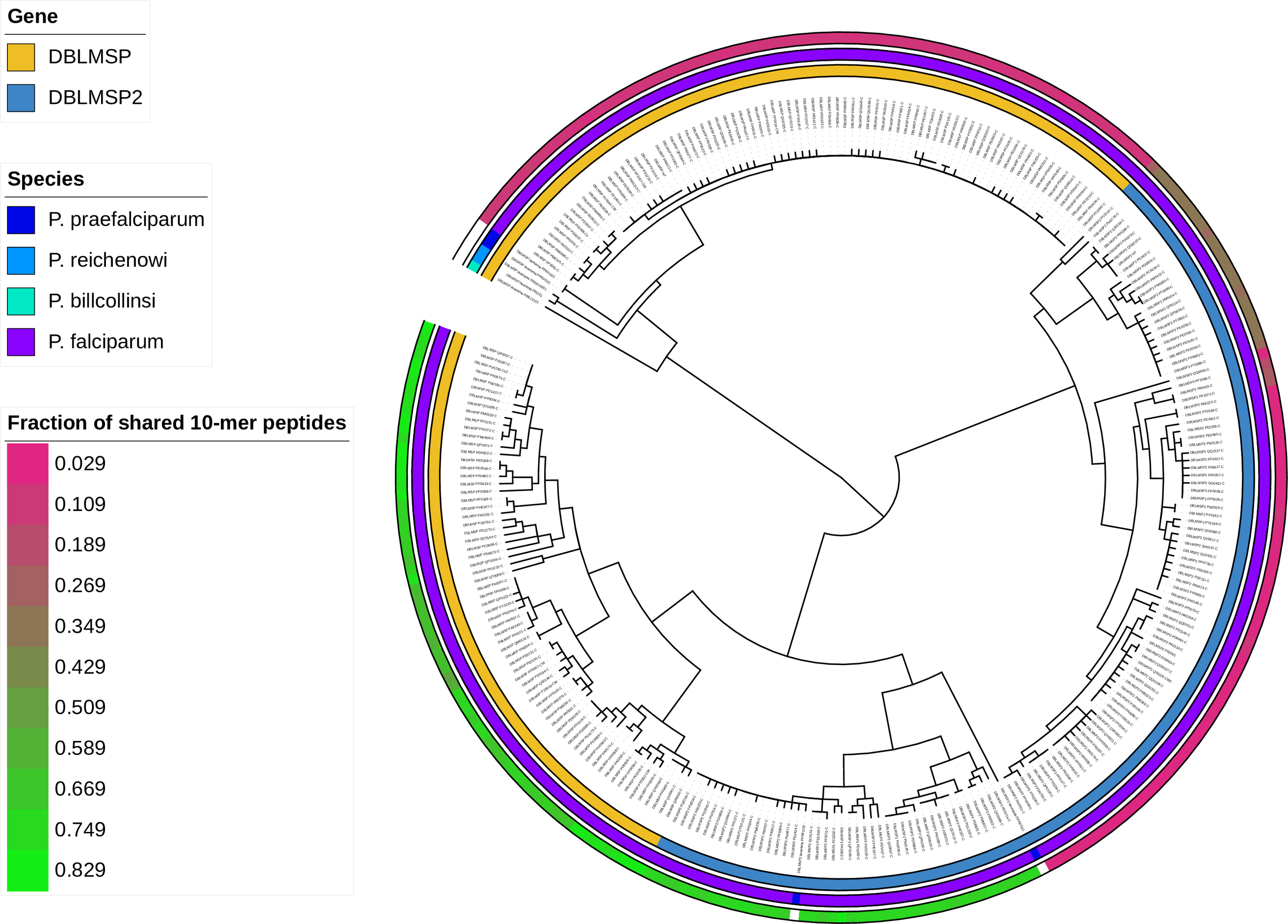

Supplement: S11 Fig — The 2 innermost rings show the gene and species of origin (as in Fig 2), and the outermost ring measures the level of sequence sharing between the 2 genes (see definition in the text). The data and code to generate this Figure can be found at https://zenodo.org/doi/10.5281/zenodo.7677547. (TIF) [file pbio.3002507.s012.tif]

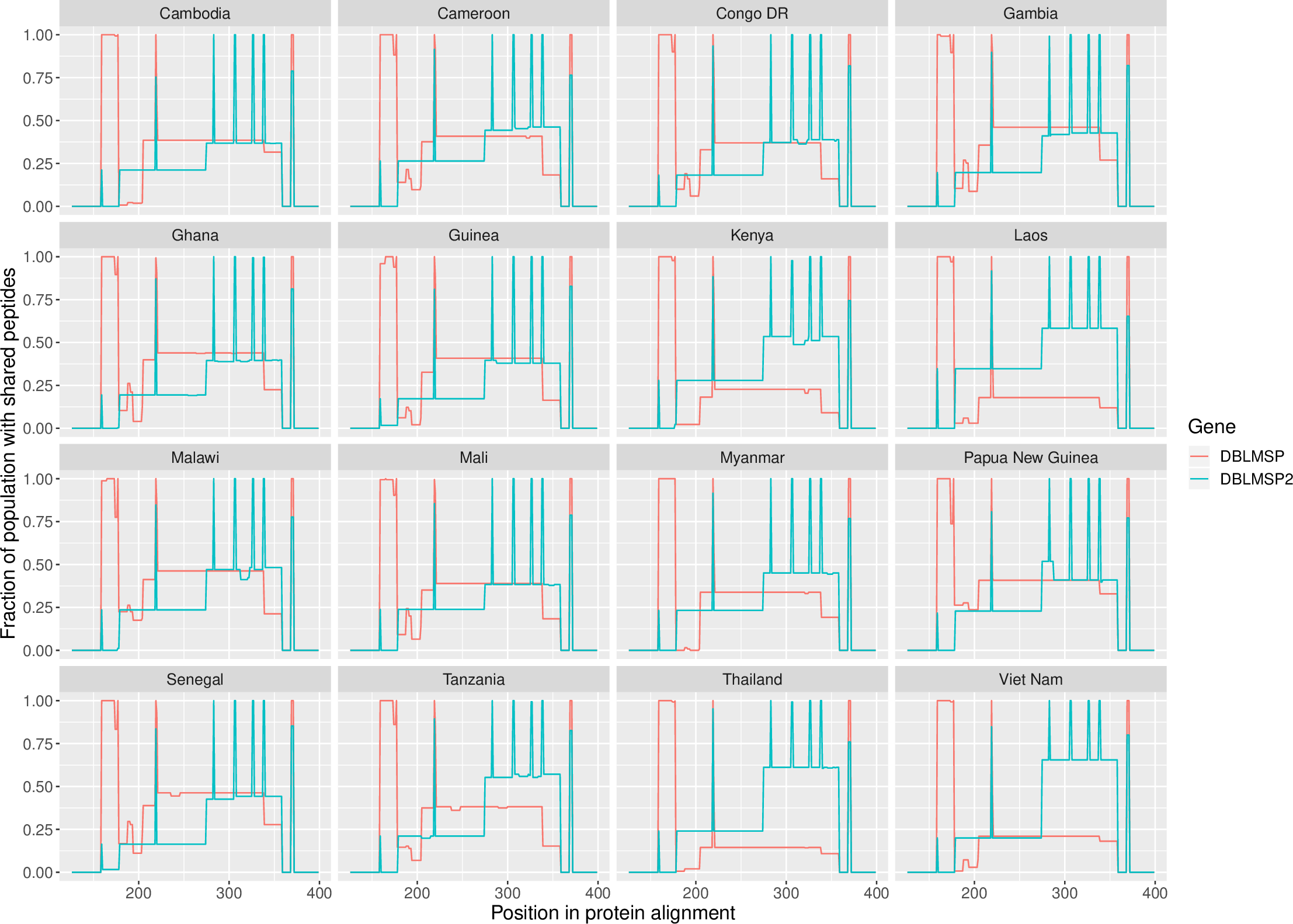

Supplement: S12 Fig — The frequency of shared peptides (y-axis), at each position (x-axis), is shown for the 16 countries with more than 50 sequences. Colour indicates frequency in each gene. Shared peptides are found at high frequencies, between 25% and 50%, across all countries. By extension, private peptides are also frequent, as any nonshared peptide is a private peptide. Values of zero, at the left and right ends of the x-axis, show the diverged flanks of the region, while values of one correspond to peptides that are always identical in both genes, i.e., where any mutations are likely eliminated by selection. On the left-hand side of the plots, DBLMSP2 displays a region with low shared peptide frequency across all countries, indicating this region has almost fully diverged between DBLMSP and DBLMSP2. The data and code to generate this Figure can be found at https://zenodo.org/doi/10.5281/zenodo.7677547. (TIF) [file pbio.3002507.s013.tif]

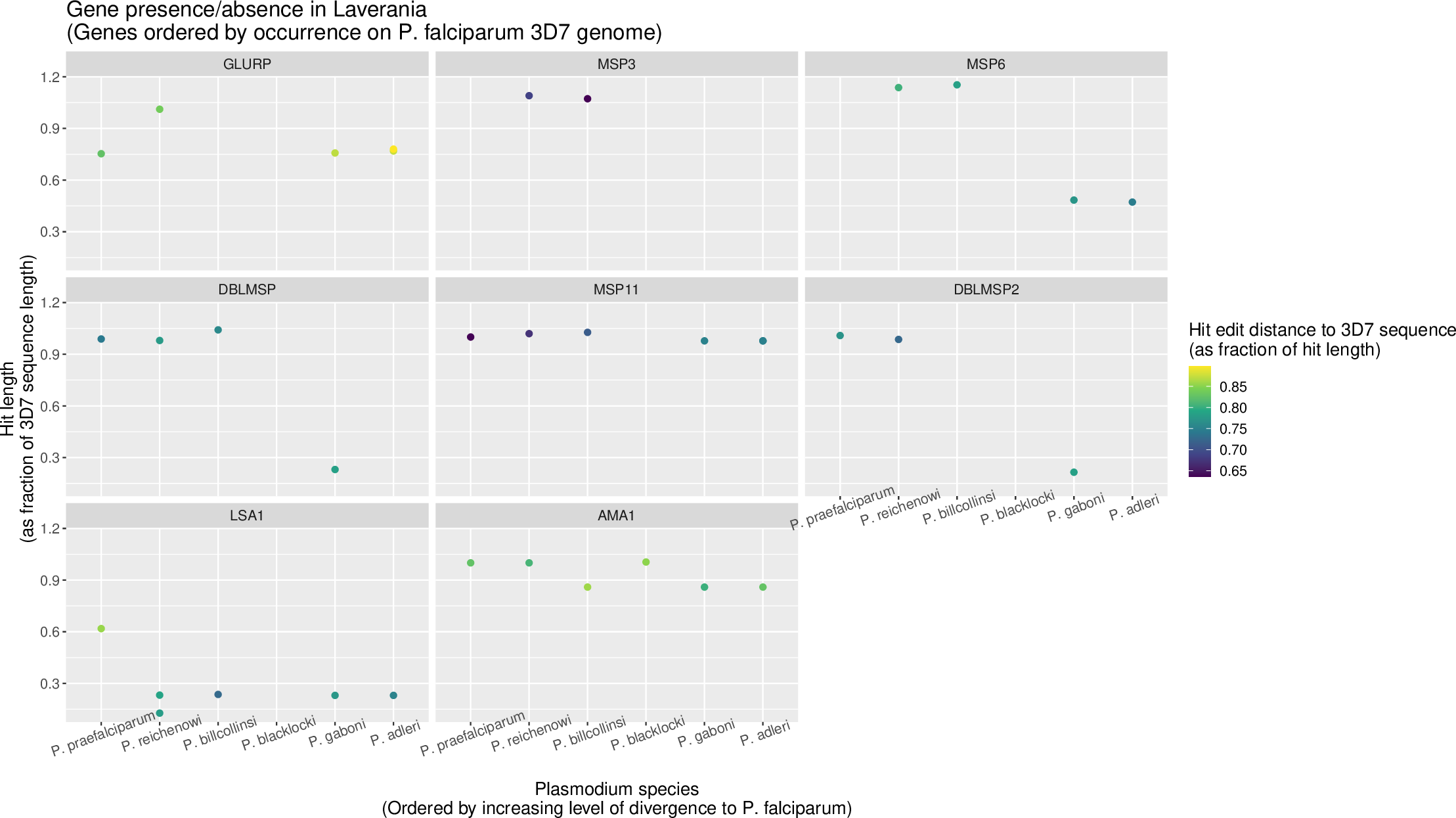

Supplement: S13 Fig — For each P. falciparum gene (panels), orthologs were searched for using minimap2 (preset: “-x asm20”). The y-axis shows the length of each hit normalised by the length of the P. falciparum gene sequence, and hits are coloured by % identity between query sequence and target in each Laverania assembly. The first 7 panels show genes occurring contiguously in a 40-kbp stretch of chromosome 10 on the P. falciparum 3D7 reference genome, and AMA1 was added as we expected it to be well conserved and found in single-copy. AMA1 could indeed be found in full length across all 6 Laverania assemblies, as was MSP11, a gene located in-between DBLMSP and DBLMSP2. We note that many genes are missing in P. blacklocki; this is most likely due to a restrictive form of whole-genome amplification prior to sequencing, which the original authors noted led to missing core genes in the resulting assembly [17]. The data and code to generate this Figure can be found at https://zenodo.org/doi/10.5281/zenodo.7677547. (TIF) [file pbio.3002507.s014.tif]

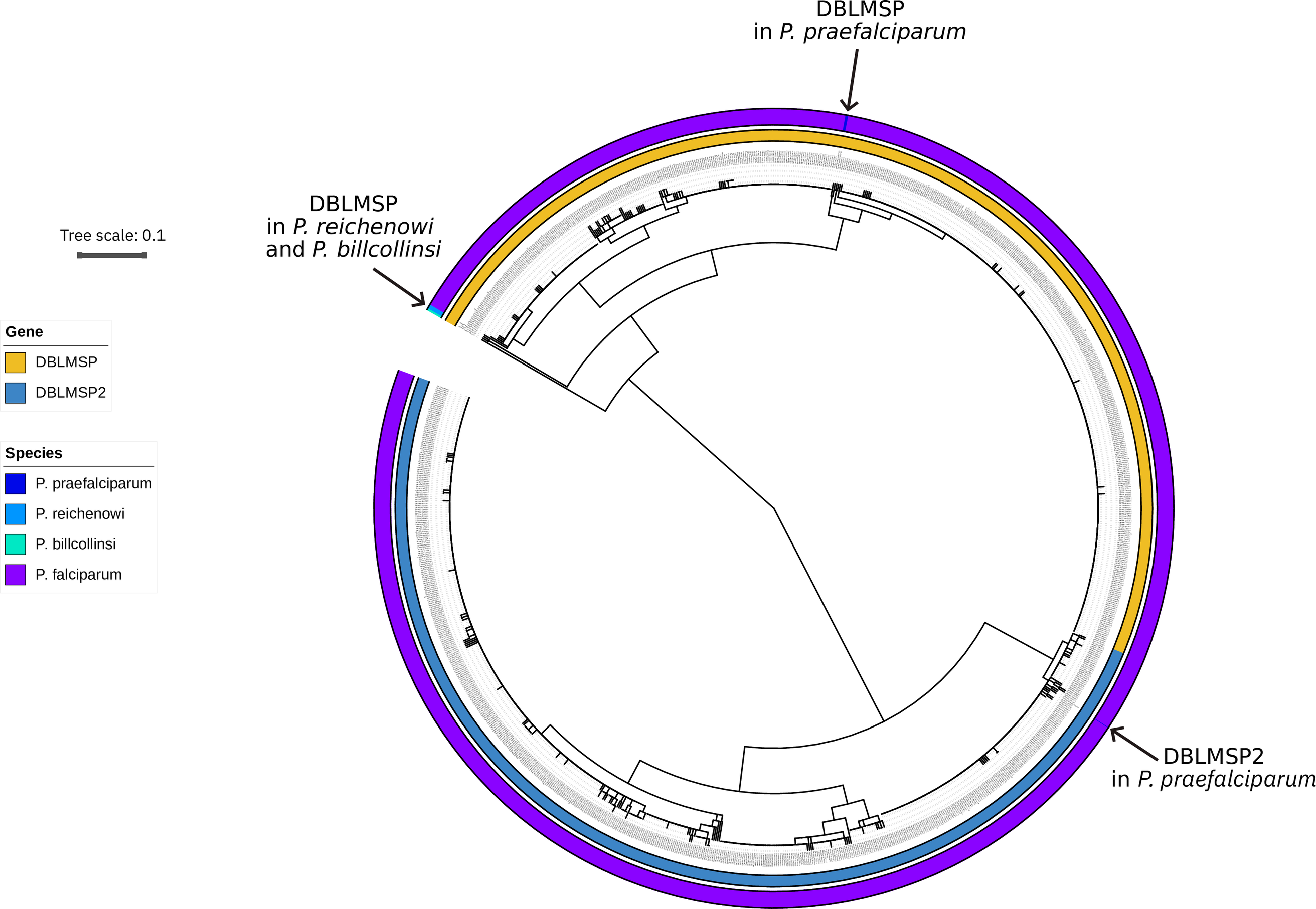

Supplement: S14 Fig — This Figure is the same as Fig 2, except that the tree was built from all unique DBLMSP1/2 full-length protein sequences, and not just of the DSR. While DBLMSP sequences from P. reichenowi and P. billcollinsi are outgroups in the clade of DBLMSP alleles, the sequences of DBLMSP and DBLMSP2 from P. praefalciparum fall nested within clades of P. falciparum alleles. This is consistent with a recent radiation of P. falciparum from a P. praefalciparum-like ancestor. DBLMSP2 is absent in P. billcollinsi and not shown in the tree for P. reichenowi as it is pseudogenised. The data and code to generate this Figure can be found at https://zenodo.org/doi/10.5281/zenodo.7677547. (TIF) [file pbio.3002507.s015.tif]

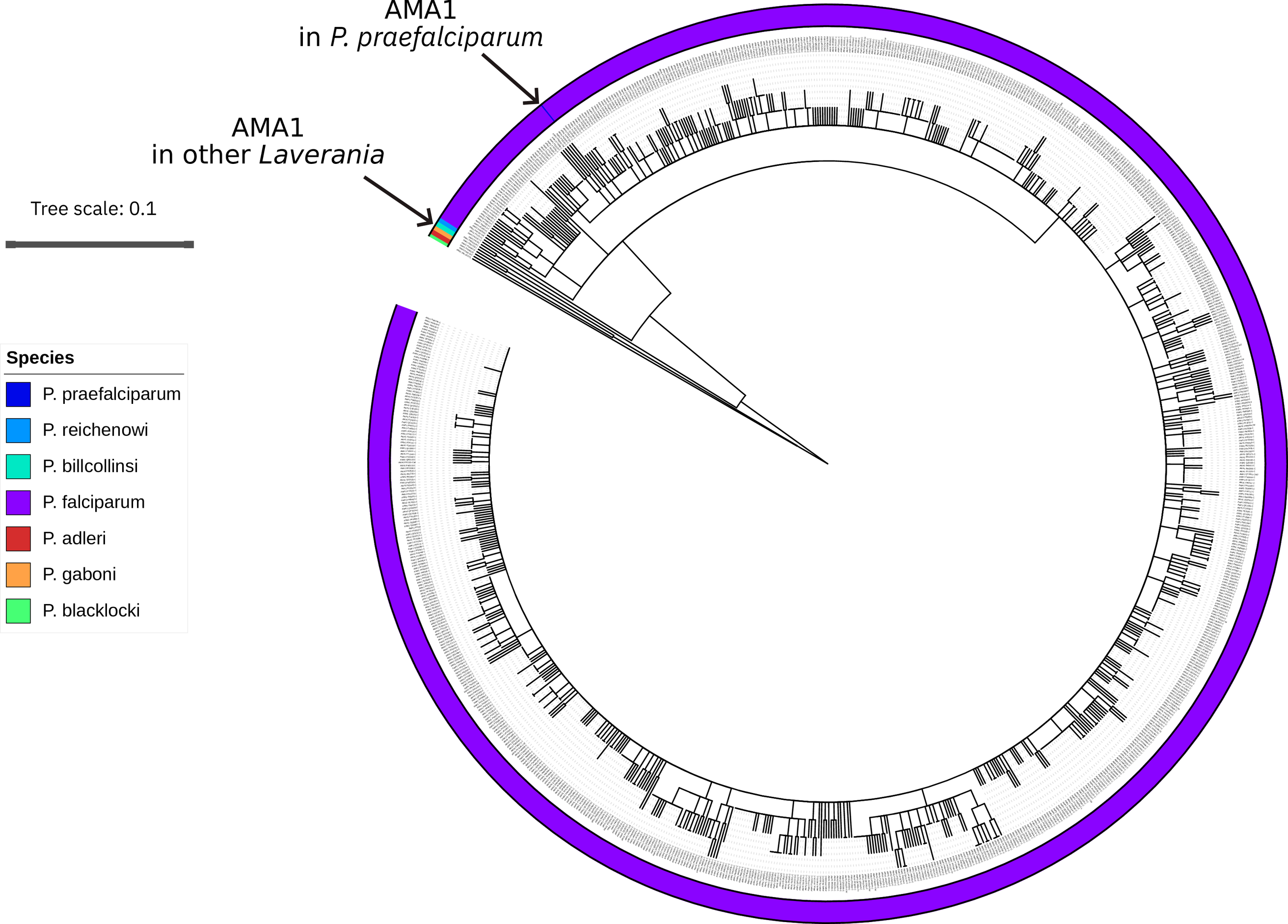

Supplement: S15 Fig — As for S14 Fig above, the orthologous sequences from P. praefalciparum falls inside a P. falciparum clade, consistent with a recent radiation of P. falciparum from a P. praefalciparum-like ancestor, while orthologs from the other Laverania species occur as outgroups to P. falciparum alleles. The data and code to generate this Figure can be found at https://zenodo.org/doi/10.5281/zenodo.7677547. (TIF) [file pbio.3002507.s016.tif]

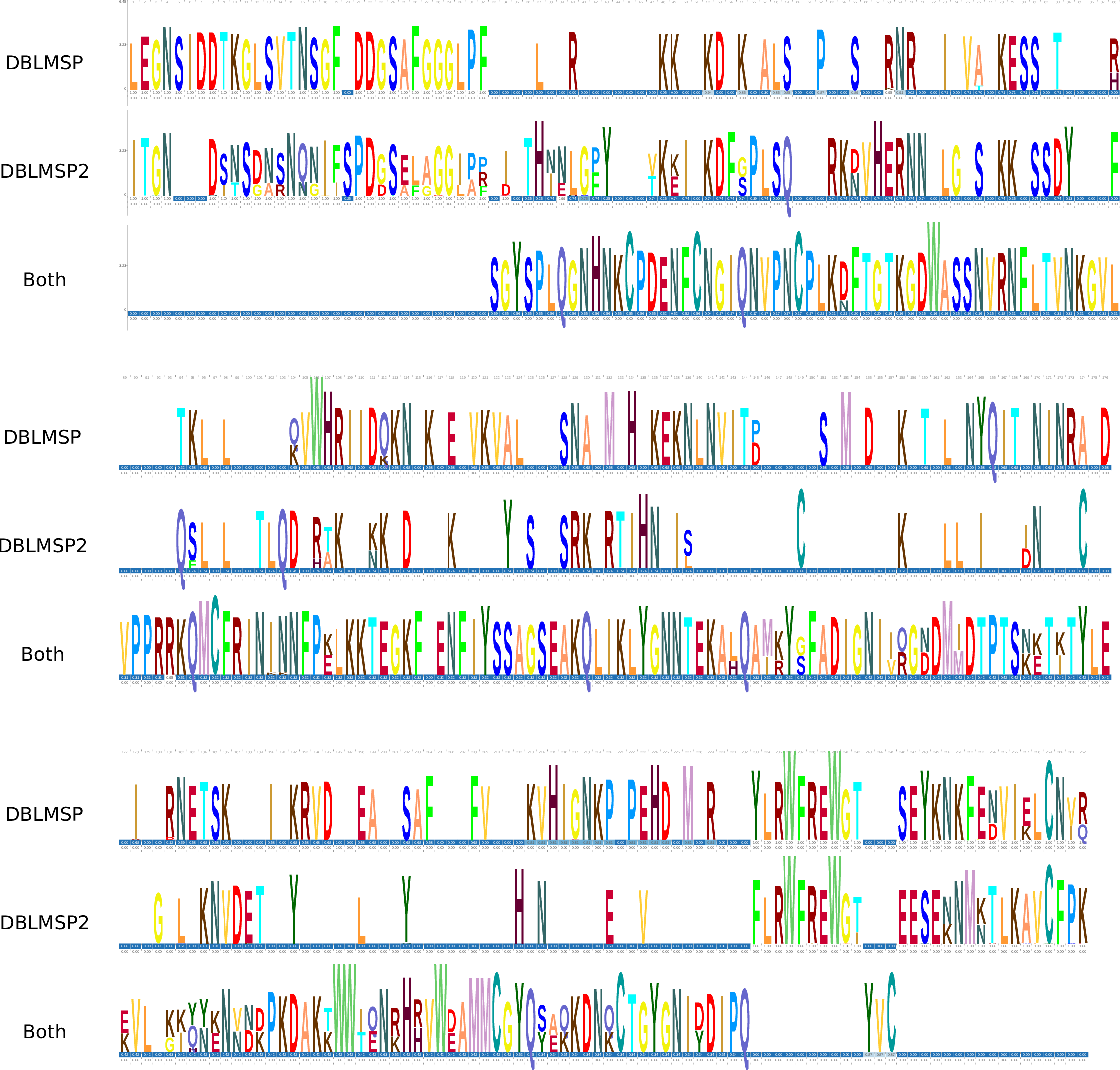

Supplement: S16 Fig — One logo was produced for peptides found only in DBLMSP (top panel), only in DBLMSP2 (middle panel), and found on both genes (lower panel, labelled “Both”). The 3 tracks are broken into segments for visual clarity. At each position, observed amino acids are shown, with letter height proportional to amino acid frequency. In-between diverged N- and C-terminal regions, there is mostly 1 prototypical private sequence for each gene (first 2 tracks) and 1 prototypical shared sequence (or 2, in the C-terminal half of the protein domain). The data and code to generate this Figure can be found at https://zenodo.org/doi/10.5281/zenodo.7677547. (TIF) [file pbio.3002507.s017.tif]

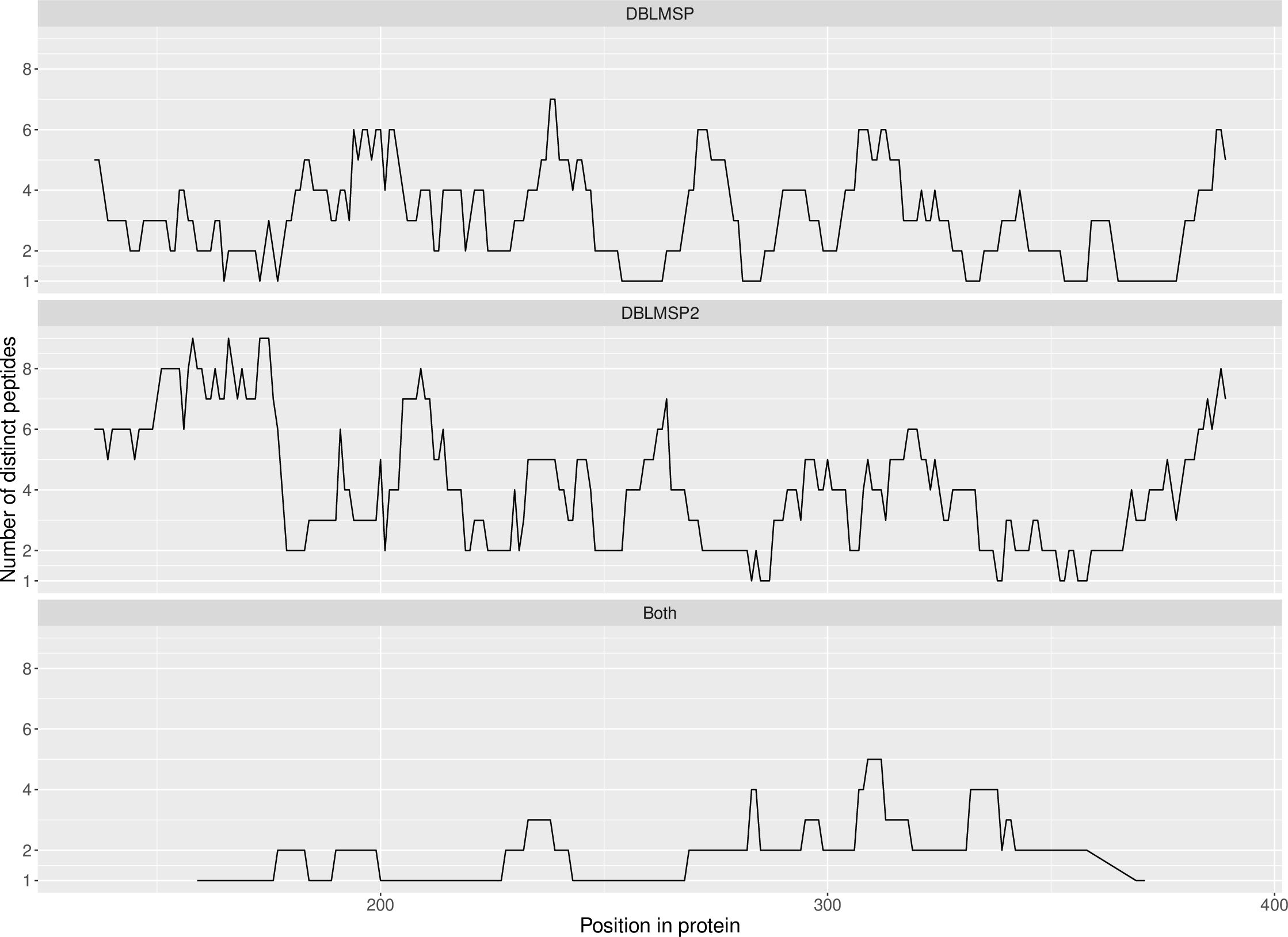

Supplement: S17 Fig — For the 2 private and 1 shared MSAs, containing peptides only found in DBLMSP (top panel), only found in DBLMSP2 (middle panel), or both (lower panel), the total number of distinct peptide 10-mers at each position is shown. Mostly 1 to 4 peptides were observed at each position in the shared category, while 2 to 6 were observed on each gene only. This figure complements S16 Fig, which shows that mostly two 10-mer peptides occur in each gene with high frequency—here, total number is shown, regardless of frequency. The data and code to generate this Figure can be found at https://zenodo.org/doi/10.5281/zenodo.7677547. (TIF) [file pbio.3002507.s018.tif]

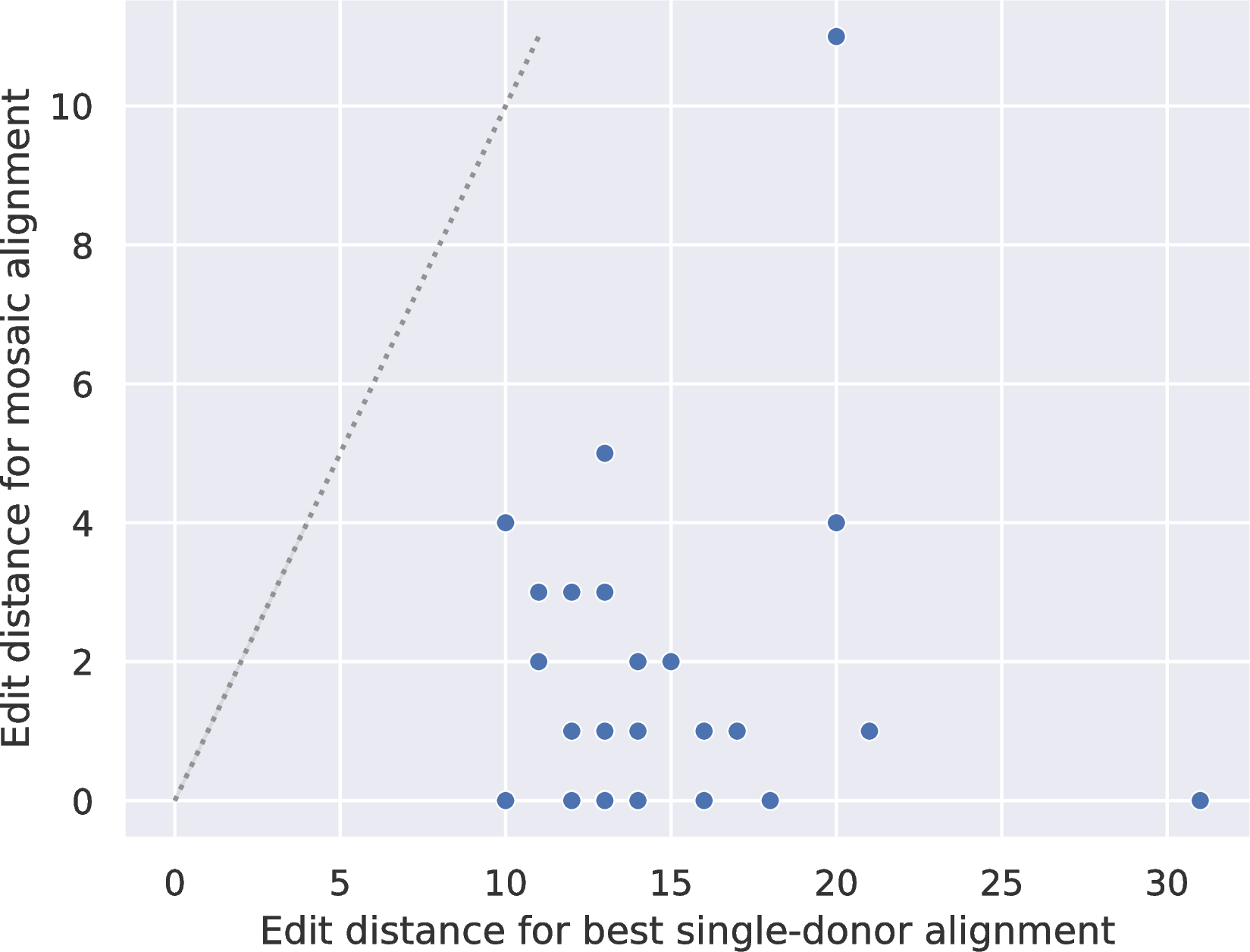

Supplement: S18 Fig — The blue dots show, for each target, its edit distance to the path of donors inferred by MosaicAligner (y-axis) and the edit distance to the single closest donor (x-axis). The grey dotted line shows y = x. All inferences reduce edit distances to the single closest donor, supporting adding recombination breakpoints to the alignment. The data and code to generate this Figure can be found at https://zenodo.org/doi/10.5281/zenodo.7677547. (TIF) [file pbio.3002507.s019.tif]

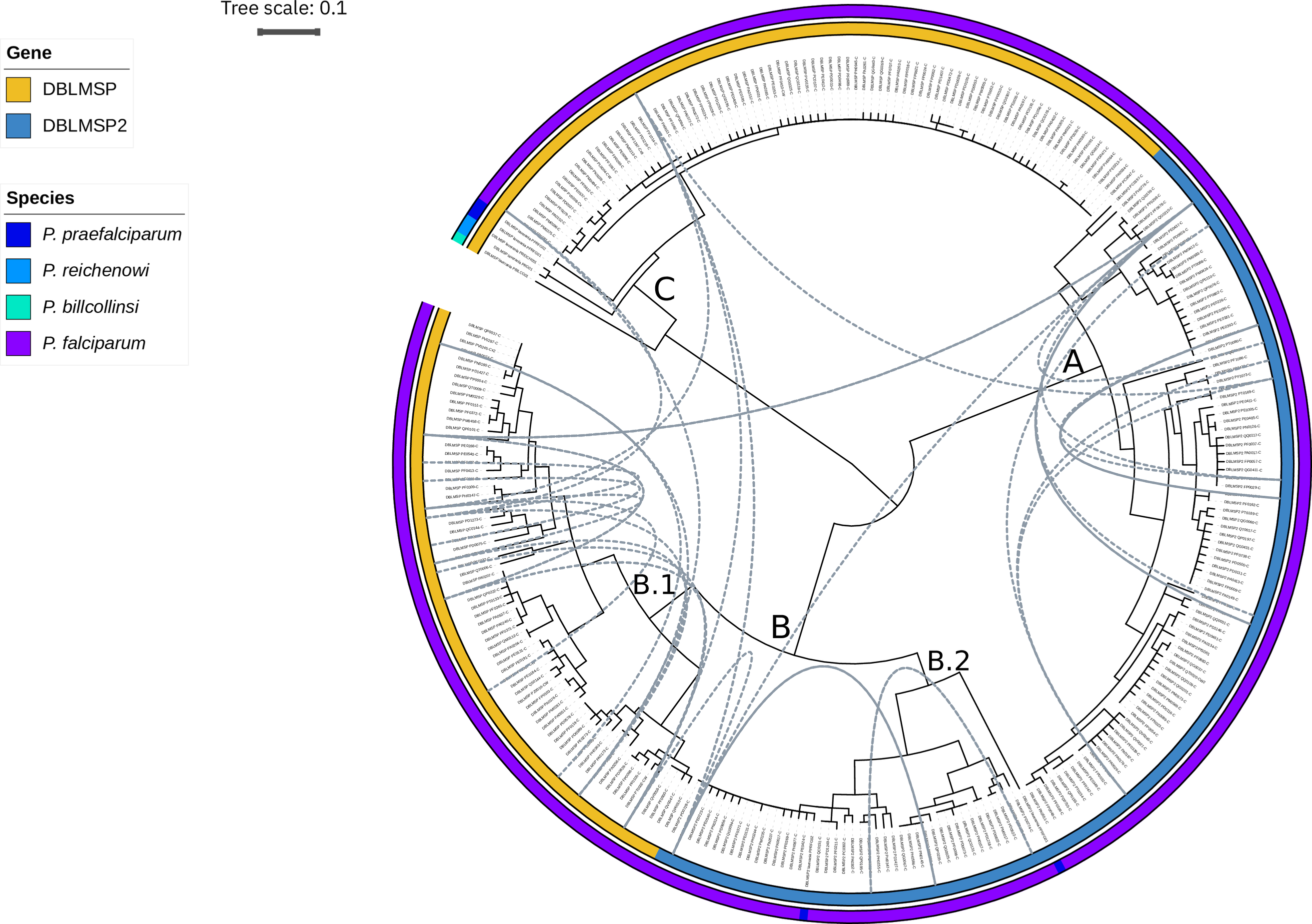

Supplement: S19 Fig — The same clustering tree as in Fig 2 of the main text is shown, with the addition of dotted lines that connect 2 sequences if they were inferred to have recombined at some point in the past (see main text and Methods for how). Most recombination events occurred within the main lineages of the tree (e.g., within A, or within B.1), but a few events also occurred between highly diverged lineages of the tree (e.g., between C and A, or C and B.2). The data and code to generate this Figure can be found at https://zenodo.org/doi/10.5281/zenodo.7677547. (TIF) [file pbio.3002507.s020.tif]

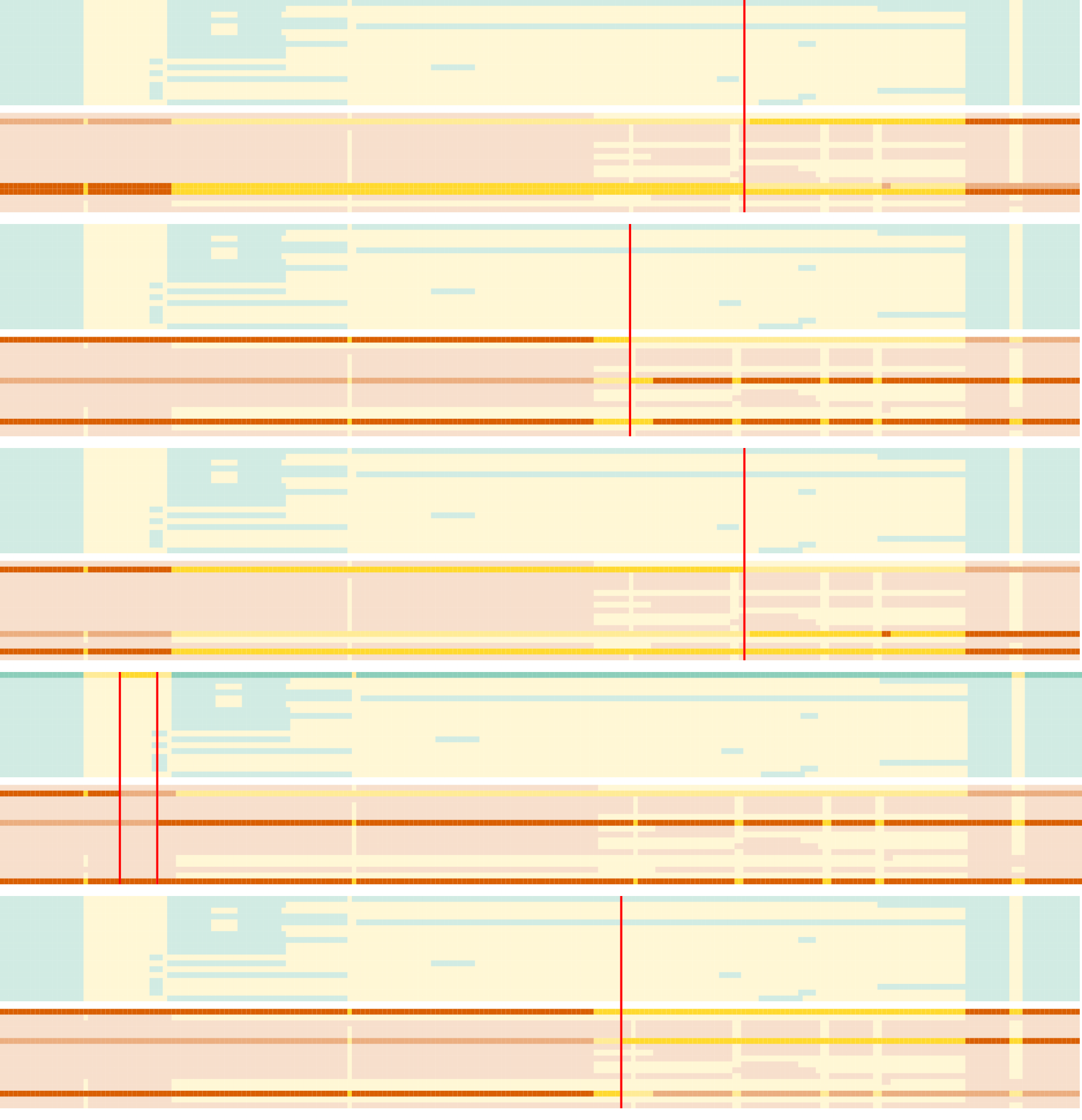

Supplement: S20 Fig — Five different recombinations are shown inside matrices, where, as in Fig 4 of the main text, each matrix depicts the mosaic alignment of 1 target sequence to the panel of 35 sequences. Sequences from DBLMSP (top) and DBLMSP2 (bottom) are separated by a white horizontal strip. Each cell is coloured by whether the size-10 peptide centred at that position occurs only in DBLMSP (blue-green), only in DBLMSP2 (orange), or in both (yellow). Recombinations mostly occur within the private DBLMSP2 lineage (all donors are mostly orange) and within the shared DBLMSP2 lineage (all donors are mostly yellow). In the last panel, the target is a recombinant of a highly private and a highly shared sequence. The data and code to generate this Figure can be found at https://zenodo.org/doi/10.5281/zenodo.7677547. (TIF) [file pbio.3002507.s021.tif]

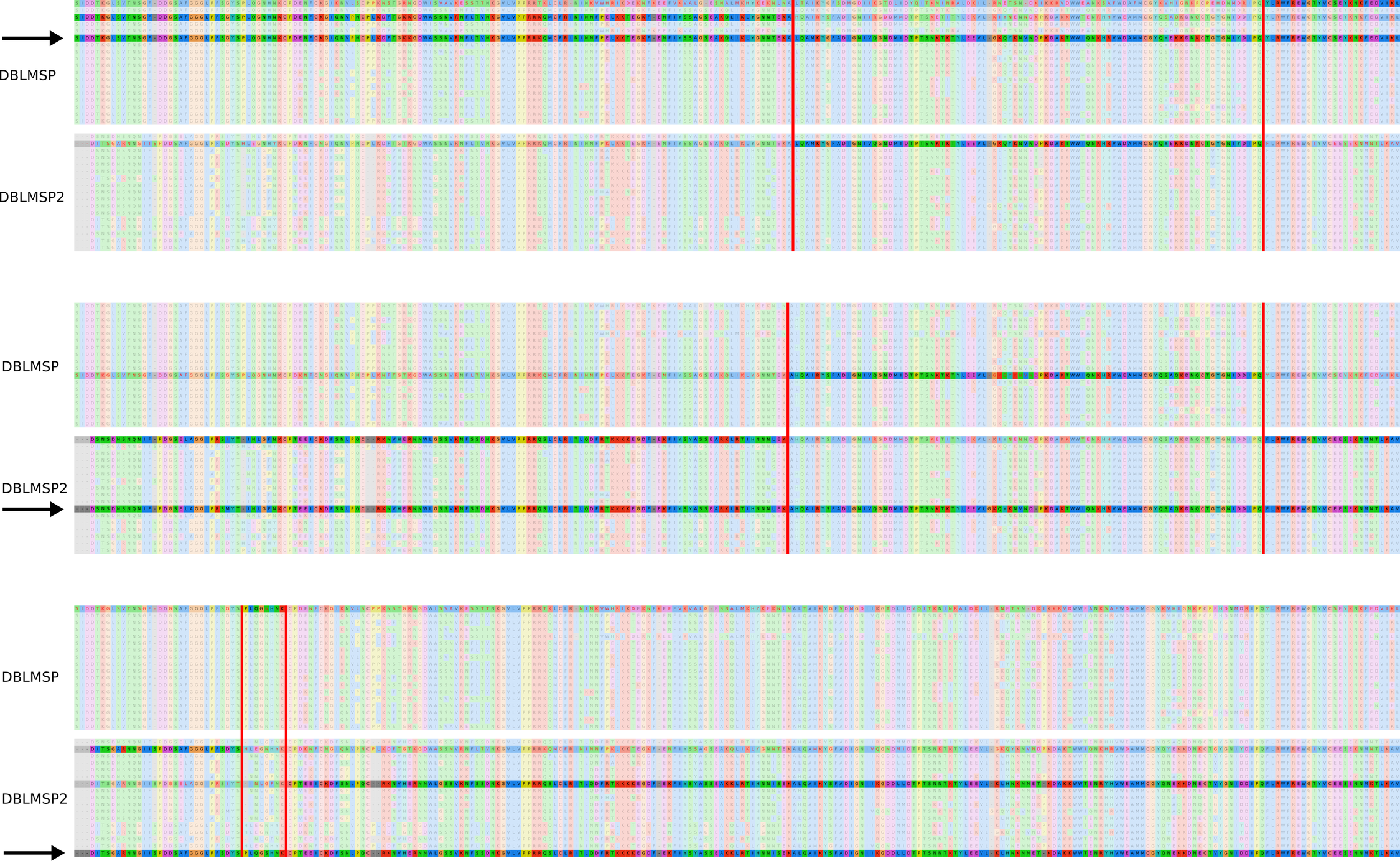

Supplement: S21 Fig — In each panel, 2 recombination breakpoints can be seen (red vertical lines). The target sequence is the fully opaque one (along its entire length; indicated with a black arrow), and the donor sequences (those the target aligns to) are shown as highlighted in places they match the target, and less opaque where they do not. In each panel, the target aligns to donors across the 2 different genes, consistent with gene conversion between the genes. The data and code to generate this Figure can be found at https://zenodo.org/doi/10.5281/zenodo.7677547. (TIF) [file pbio.3002507.s022.tif]

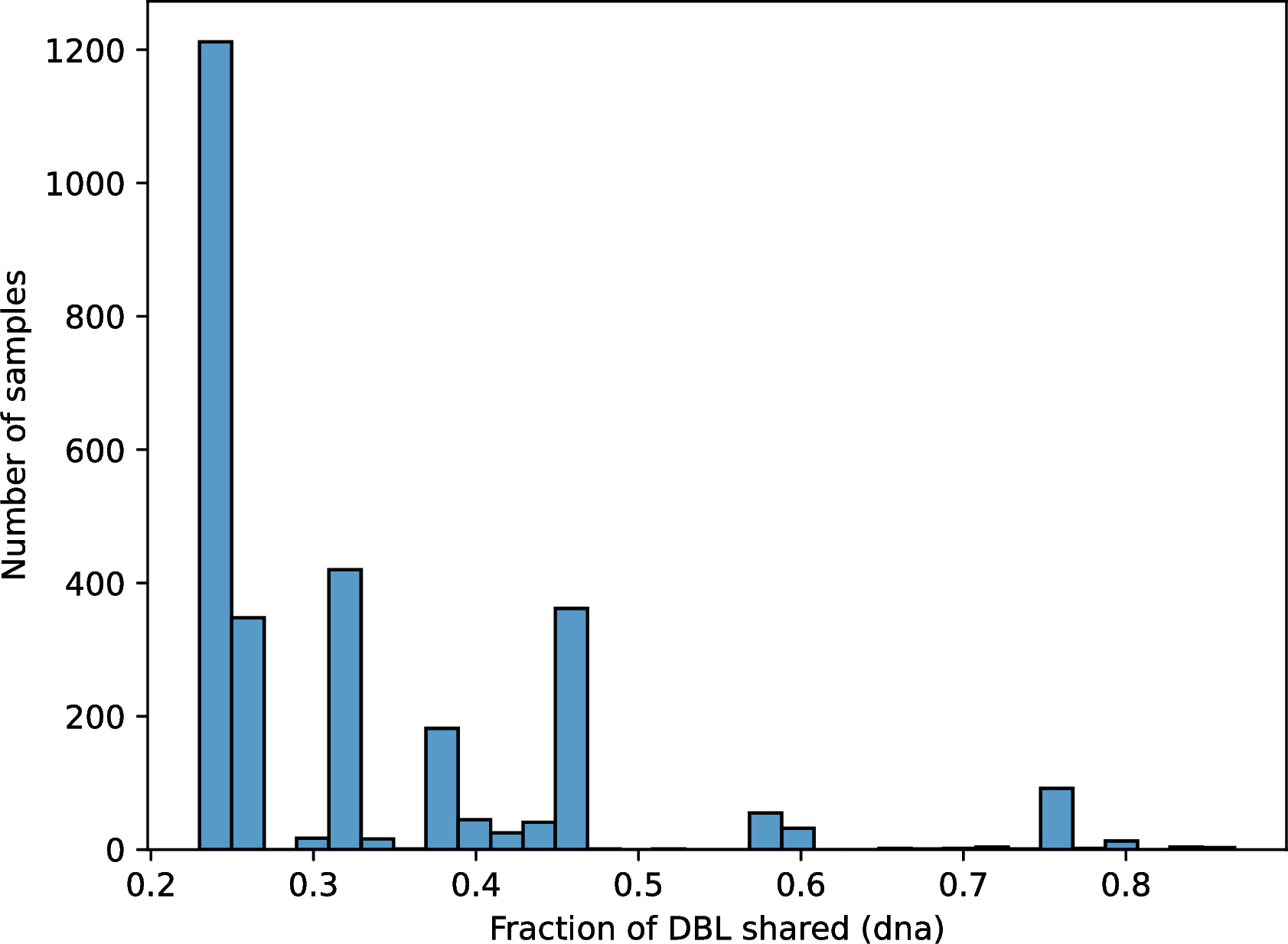

Supplement: S22 Fig — For all samples in which both DBLMSP1/2 sequences were confidently resolved, the DNA sequences of DBLMSP and DBLMSP2 within a single genome were aligned and the fraction of identical codons in the DSR recorded. Most samples have quite low identity levels (e.g., 0.2 up to 0.4), and a minority of samples have high identity levels, defined as >0.5 identity. The latter samples are illustrated in Fig 4 of the main text. The data and code to generate this Figure can be found at https://zenodo.org/doi/10.5281/zenodo.7677547. (TIF) [file pbio.3002507.s023.tif]

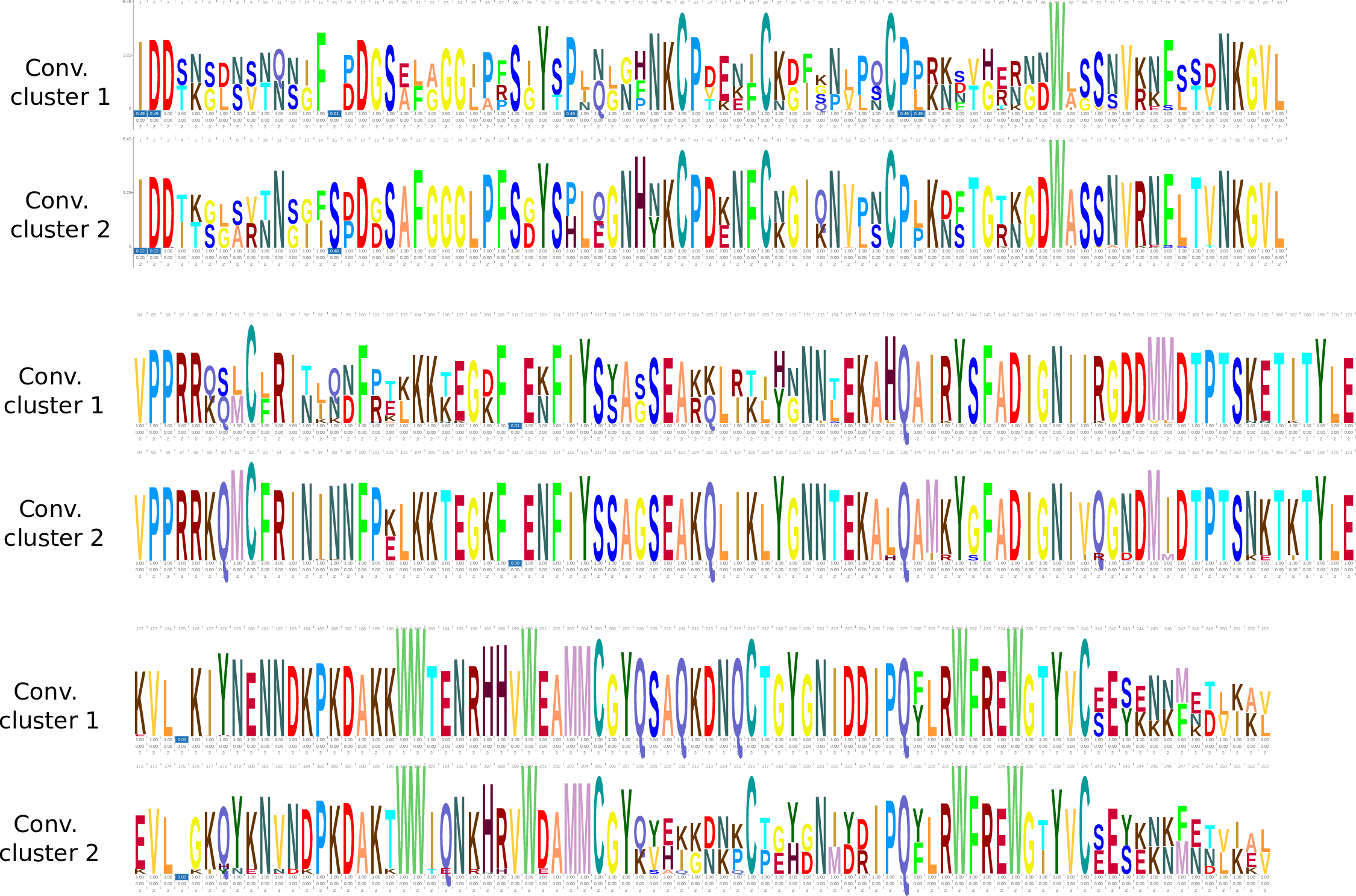

Supplement: S23 Fig — One logo was produced for each of the 2 conversion events in Fig 4 of the main text, and each logo split into 3 portions for visual clarity. While at many positions, the sequences in each conversion event overlap, each is enriched for different amino acids, and some positions have entirely different amino acids. This supports a distinct evolutionary trajectory for each event and thus at least 2 distinct gene conversion events having occurred in DBLMSP1/2. The data and code to generate this Figure can be found at https://zenodo.org/doi/10.5281/zenodo.7677547. (TIF) [file pbio.3002507.s024.tif]

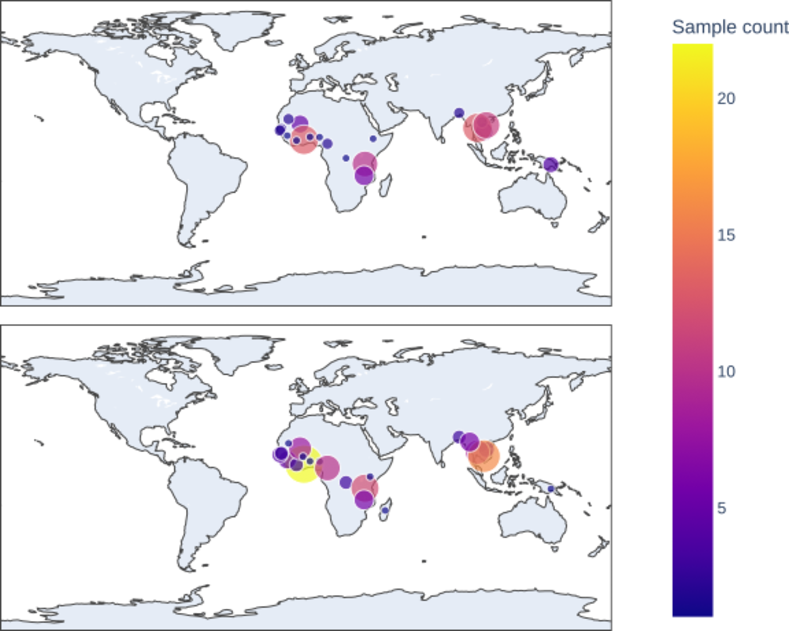

Supplement: S24 Fig — The 2 panels correspond to the 2 gene conversion events identified in Fig 4 of the main text (in the same order). In each panel, the number of samples in each geographical region is shown, both through the size and colour of dots. For both conversion events, samples are geographically widespread, occurring across west and east Africa and Southeast Asia. The base map comes from the freely distributed python package “plotly” (function “plotly.express.scatter_geo”), under an MIT licence: https://github.com/plotly/plotly.py. The data and code to generate this Figure can be found at https://zenodo.org/doi/10.5281/zenodo.7677547. (TIF) [file pbio.3002507.s025.tif]

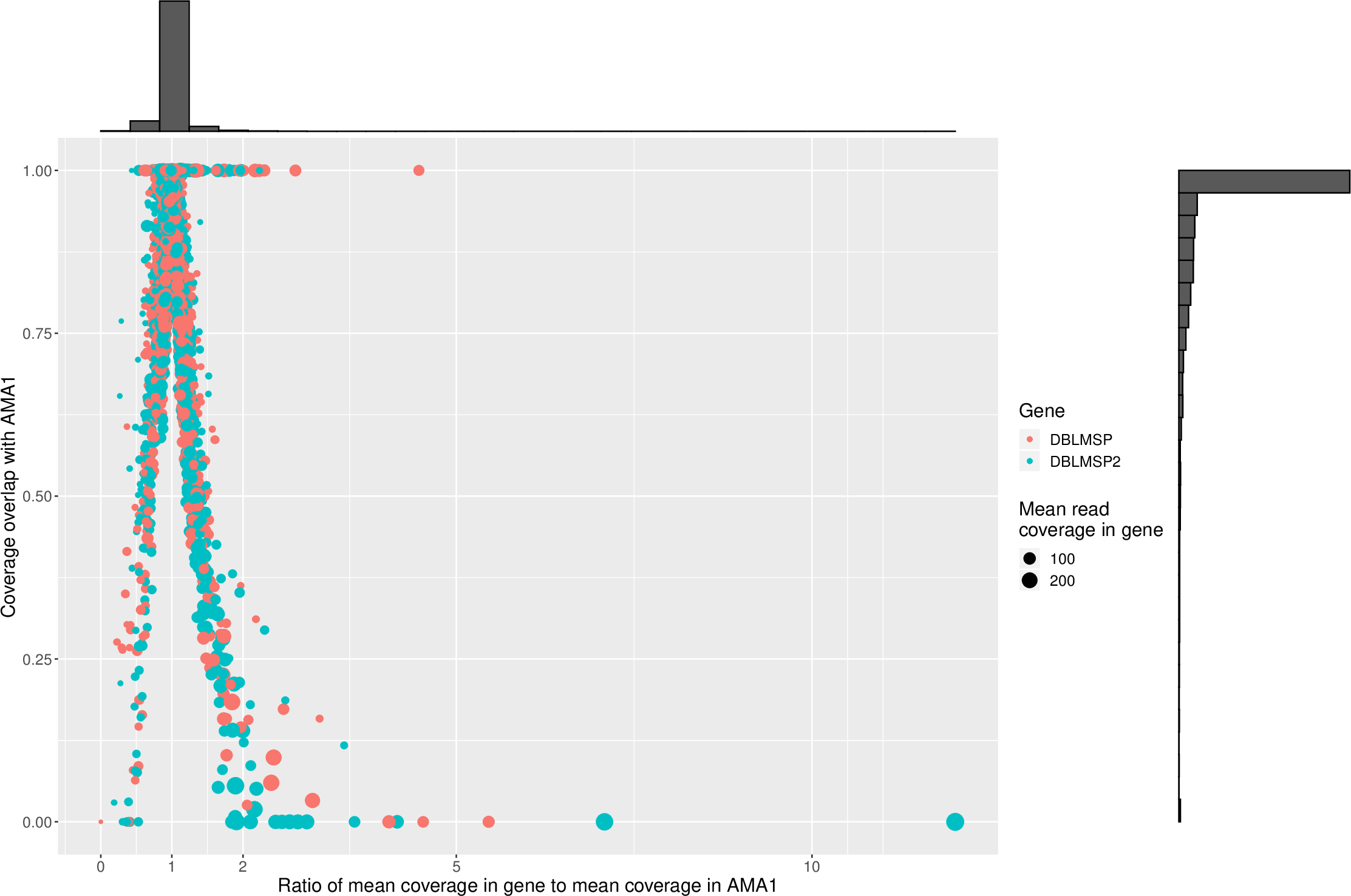

Supplement: S25 Fig — For all 3,589 analysis-set samples, the mean and standard deviation (std) of the per-base read coverage of reads realigned the “induced reference” (S5 Fig, panel b) was measured in genes DBLMSP, DBLMSP2, and AMA1. For each gene, we produced a coverage interval {mean– 2 * std, mean + 2 * std}, which we consider a “plausible range” of gene-level coverage. The x-axis shows the ratio of the mean coverage in DBLMSP1/2 to that in AMA1, a gene that we assume to be single-copy in all samples. The marginal distribution histogram is shown on top. Most samples have a ratio of 1, and some have ratios <0.5 or >2, indicating possible copy-number changes. The y-axis shows the fraction of the DBLMSP or DBLMSP2 coverage interval overlapped by the AMA1 coverage interval. Most samples have totally overlapping intervals (marginal distribution on right-hand side). Small overlap values indicate more likely true differences in coverage. Of the 6,123 analysed (“confidently resolved”) DBLMSP and DBLMSP2 sequences, 31 had a fold-coverage >2 and an overlap <0.5 (bottom-right of plot), indicating putative duplication. Three of these overlapped with samples with evidence of gene conversion and were filtered out in that analysis (Fig 4 of the main text). The data and code to generate this Figure can be found at https://zenodo.org/doi/10.5281/zenodo.7677547. (TIF) [file pbio.3002507.s026.tif]

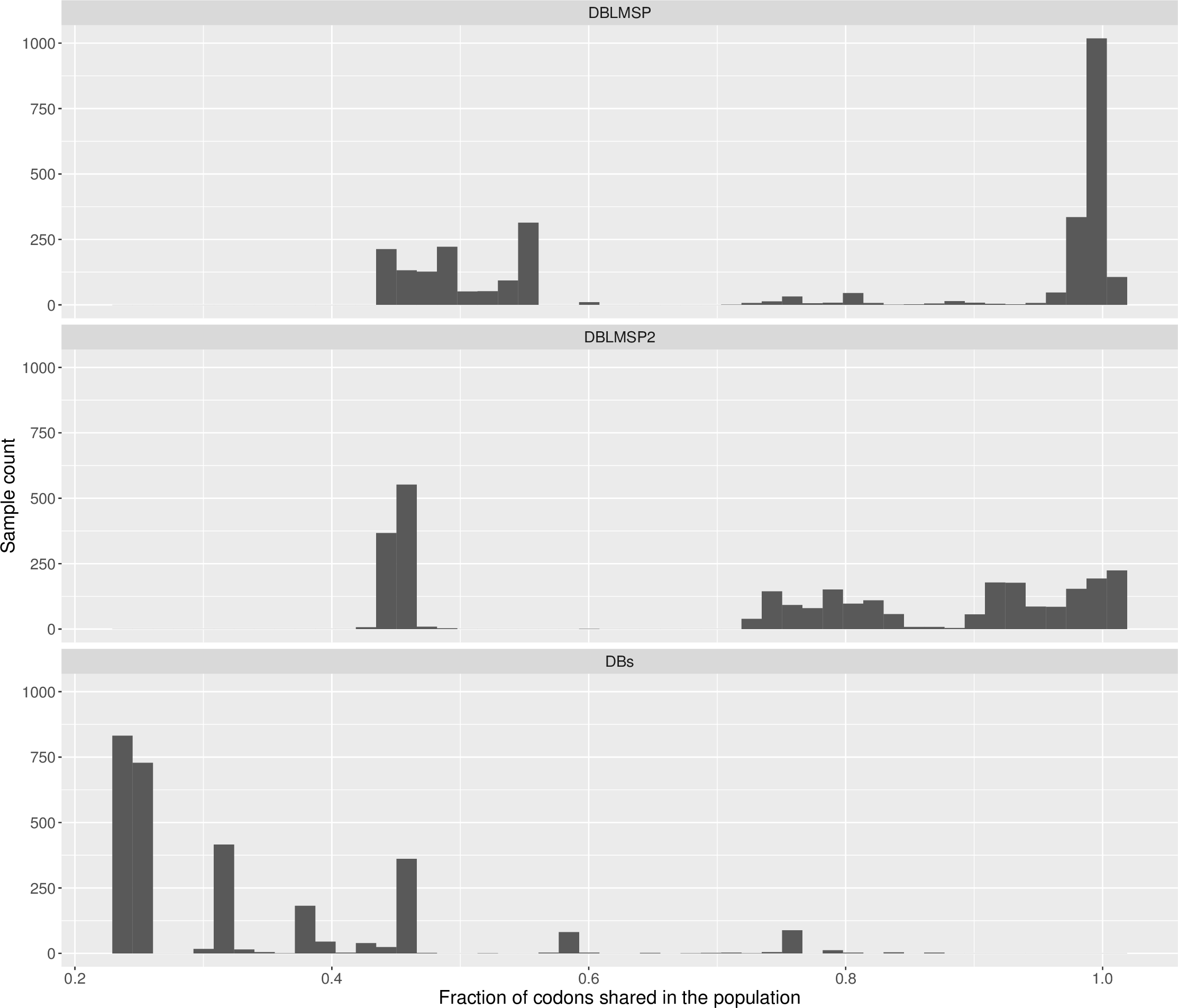

Supplement: S26 Fig — The 2 first panels measure, for each of DBLMSP and DBLMSP2, the percent codon identity of 2,882 randomly chosen gene pairs and is a measure of sequence diversity. The third panel shows the percent codon identity between DBLMSP and DBLMSP2 across all 2,882 samples where they were confidently resolved and is a measure of sequence divergence. Between-gene divergence exceeds within-gene diversity (lower codon identity across genes than within genes). The data and code to generate this Figure can be found at https://zenodo.org/doi/10.5281/zenodo.7677547. (TIF) [file pbio.3002507.s027.tif]

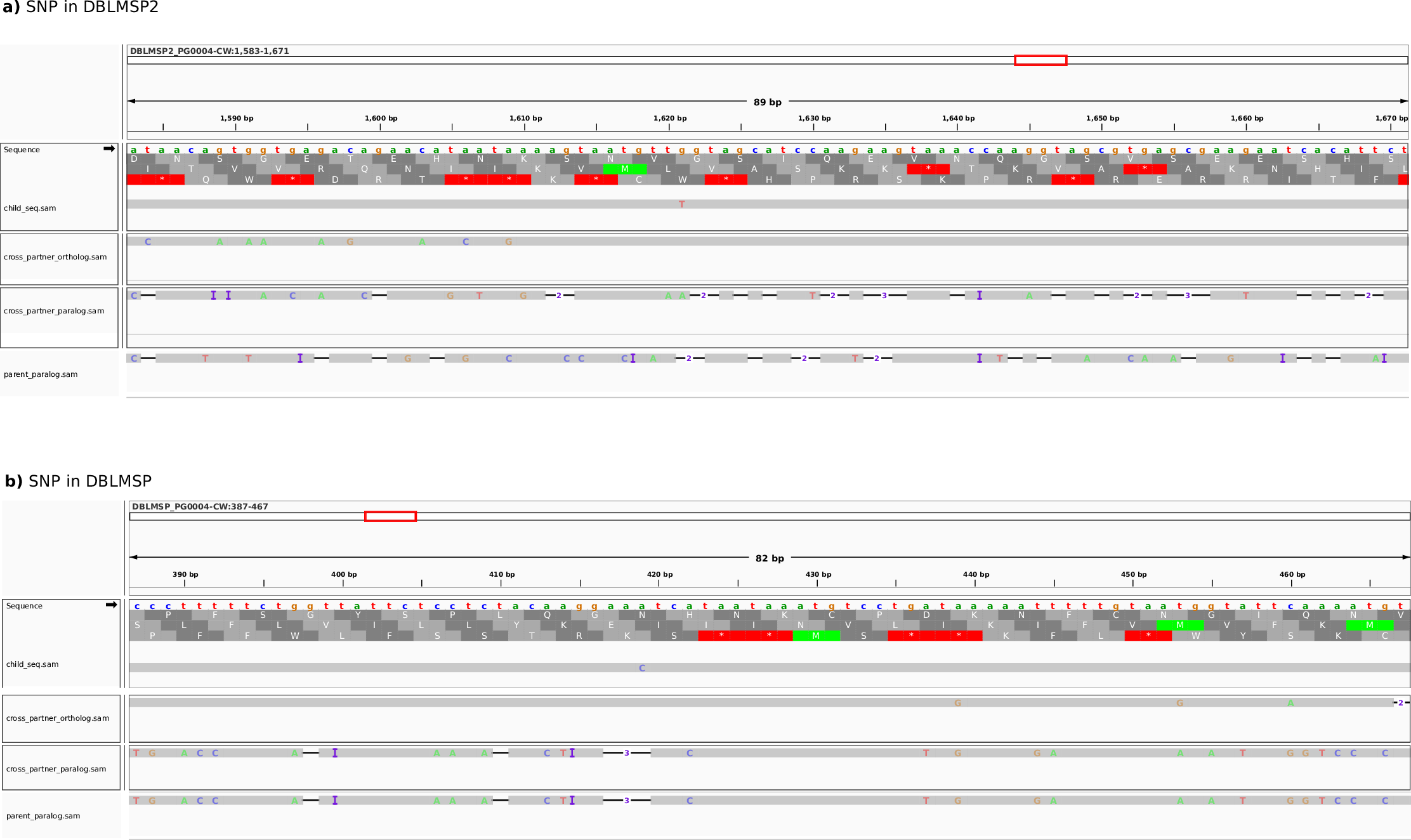

Supplement: S27 Fig — In 1 progeny sample from genetic cross HB3xDd2, 2 SNPs were identified in DBLMSP1/2, one in each gene (panel a: DBLMSP2, panel b: DBLMSP). In both panels, the top track shows the parent gene sequence (HB3), and 4 subsequent tracks are shown below, each representing 1 different aligned sequence (grey horizontal bars). The first aligned sequence is the child sample gene sequence, showing a single SNP difference to the parent. To confirm these were spontaneous mutations and not single-base gene conversions from a homolog, 3 homologous sequences that could have been conversion donors were aligned to the parent: the orthologous sequence from the other cross parent (Dd2, second track), and the paralogous sequences from both parents (third and fourth tracks). No matches to these at the SNP positions can be seen. The data and code to generate this Figure can be found at https://zenodo.org/doi/10.5281/zenodo.7677547. (TIF) [file pbio.3002507.s028.tif]
