# Supplementary material for: Role for gene conversion in the evolution of cell-surface antigens of the malaria parasite Plasmodium falciparum
Source: PLoS Biol. 2024 Mar 7;22(3):e3002507. doi: 10.1371/journal.pbio.3002507 (PMC10919680; doi:10.1371/journal.pbio.3002507)
Supplement: S1 Table — Each tool’s approach and main strengths are summarised. “Specific” refers to low false-positive rates in variant calling, and “Sensitive” to high true-positive rates. (DOCX) [file pbio.3002507.s029.docx]

| **Tool** | **Method** | **Strengths** |
| --- | --- | --- |
| Cortex | Global assembly | Specific; can assemble diverged regions |
| Octopus | Pileup analysis and local assembly | Sensitive and specific; good for SNPs, indels and repeats |
| gramtools (adjudication) | Genome graph of variation from multiple callers | Leverages different callers’ strengths |
| Gapfiller | Sequence assembly between read pairs | Can assemble diverged regions |
| gramtools (joint genotyping) | Genome graph of population variation | Allows missed call to be found |

Supplementary table 1. **Characteristics of the tools used in our new genotyping pipeline**. Each tool’s approach and main strengths are summarised. Specific refers to low false-positive rates in variant calling, and Sensitive to high true-positive rates.
