# Supplementary material for: Role for gene conversion in the evolution of cell-surface antigens of the malaria parasite Plasmodium falciparum
Source: PLoS Biol. 2024 Mar 7;22(3):e3002507. doi: 10.1371/journal.pbio.3002507 (PMC10919680; doi:10.1371/journal.pbio.3002507)
Supplement: Attachment — Submitted filename: letcher_gene_conversion - Google Docs.pdf [file pbio.3002507.s030.pdf]

Letcher et al have undertaken a study in which they have employed a novel variant detection pipeline, gramtools, which uses genome graphs to assess variants that are called with a range of different genotype callers and is less affected by divergent haplotypes than other callers. After extensive validation of this pipeline they have applied it merozoite surface proteins in P. falciparum which appear to show highly diverged haplotypes, and are subject to frequent ectopic recombination.
The improved variant calls suggest haplotypes are shared between the two MSPs, and further analysis of recombination patterns and phylogenetics strongly suggest this is a case of gene conversion.

The work is extremely thorough, comprehensive and convincing, the results are notable in the context of malaria evolution. I have little hesitation in recommending it for publication. If I have a criticism to make, it is that the validation of gramtools in the content of Pf takes up a large part of the paper and does not show significant novelty beyond Letcher 21 / Hunt 22. This detracts from the more interesting story of gene conversion between MSPs and the paper might be better served by placing this in the supplementary. This may leave space for other evolutionary implications of this result (assuming they are not being dealt with elsewhere) such as the biological effects of these haplotypes and whether metrics such as KaKs etc indicate differing selective pressures on both private and shared haplotypes.

General nitpicking below:

L113 - the range of tools appears to be cortex + octopus; these tools (and the reasons for choosing each) should be included

L127 / supplemental
Numbering and ordering of supplemental figures is either out of order or very confusing.

L181 / fig s-2.1: without context it is hard to know how high this is - how does it compare to other paralogues that are not involved in cytoadhesion?

L256: Analysis appears sound, but it took me a few reads to figure it out, so perhaps some clarification that this is an alignment between genes is needed.

L261 / Fig 5 do branch lengths derive from both the non-converted and converted regions? i.e. is there divergence between samples where gene conversion has occurred between DBs and if so how much?

L280: This figure is *very* complex and I’m not sure it adds much to the plot.

L318: This section feels like a response to a reviewer question, but IMO doesn’t add a lot to the paper.
