# Supplementary material for: Role for gene conversion in the evolution of cell-surface antigens of the malaria parasite Plasmodium falciparum
Source: PLoS Biol. 2024 Mar 7;22(3):e3002507. doi: 10.1371/journal.pbio.3002507 (PMC10919680; doi:10.1371/journal.pbio.3002507)
Supplement: Attachment — Submitted filename: round_1_revision_response_to_reviewers.pdf [file pbio.3002507.s031.pdf]

Dear Editor,

We would like to thank the reviewers for their thorough and constructive comments that led to considerable thought and improvements to our article. We respond to the reviewers in detail below, but we emphasise the following main changes:

1. As suggested by Reviewers 1 and 3, we have reconstructed sequences from the three closest *Plasmodium* species to *P. falciparum*, and added them to our analysis. This brought some updated inferences/conclusions, including that the DBLMSP/DBLMSP2 preceded the jump into humans (lines 174-175), the likely direction of one of the gene conversions (lines 288-290), and a possible human-specific sequence constraint in DBLMSP2 (discussion: lines 370-376).
2. We agree our use of a term historically used within the malaria community ("dimorphism") was confusing and restricted the accessibility to a broader readership; we have removed this term entirely except when referring to its historical meaning, in the discussion (lines 330-332). We now talk in terms of lineages instead, which is much simpler.
3. To focus the paper on our analysis of DBLMSP and DBLMSP2 sequences and their evolution, we have moved technical details on our genotyping pipeline and evaluation to the supplementary (as suggested by R2 and R3). This includes Fig. 1 of our initial submission (that described the pipeline), the technical parts of results section 1 and the methods section describing pipeline components. We find the paper now reads as less technical, making it more accessible to a broad audience.
4. We agree some figures were overly complicated, and reworked them. We moved the HMM logos (old Fig 3) to the supplement, and now have a simple Figure 2 introducing the shared and private lineages (stemming from Reviewer 2's comments), plus sequences from other *Plasmodium* species. We show, as before, evidence of recombination (Fig 3) and gene conversion (Fig. 4, with an additional new panel for clarity) and then show an updated lineage tree in Figure 5 that includes a graphical model of how sub-lineages can emerge by gene conversion. The model in Fig. 5 (panel b) and associated text are more precise than the model previously sketched in Fig. 7; we have removed the latter figure.
5. We have modified the title to no longer imply causality (no use of "drive"), focussing on evolution instead: 'Evolution of deeply-diverged lineages in two paralogous cell-surface antigens of the malaria parasite *P. falciparum*'

Reviewer #1:

This paper explores gene dimorphism in P. falciparum, by considering a pair of paralogous genes (DBLMSP and DBLMSP2) which were the subject of previous studies by some of the authors. Here' they propose a new genotyping pipeline, largely based on de novo assemblies, to obviate short-read mapping issues that occur in high-similarity paralogs. Using this method, they reconstruct the DBLMSP and DBLMSP2 sequences of several thousand samples including in the MalariaGEN dataset and, unsurprisingly, show that the pipeline is able to resolve these sequences better than the default GATK-based genotyping used by MalariaGEN. The authors then investigate sequence dimorphism in a domain of the two paralogs, and find that one of the "forms" is shared by the two genes. They also describe a number of recombination and conversion events, proposing a model for how dimorphism might have emerged as a result of population bottlenecks, speculating that this might have occurred when Pf ancestors jumped from gorilla to human hosts.

The paper is well-written and informative about the genetics of dimorphism, and the pipeline seems a valuable contribution. Generally, it would be of interest to those interested in parasite genetics, and suitable for publication. There are, however, some revision- including some conceptual adjustments- that I think are necessary.

Thank you for your review and comments, which are addressed below.

1. Major Point: The primary output data from this analysis (namely, the thousands of reconstructed nucleotide sequences of DBLMSP and DBLMSP2) are not made available as far as I can see. I believe these data will be very useful to the research community. I appreciate that the authors have gone to some effort to make the pipeline available for reproducibility, but realistically this dataset could only be replicated from scratch with the resources of a major northern institution (e.g. EMBL-EBI), to the disadvantage of researchers in malaria-endemic countries. Please make a downloadable dataset of these sequences available, labelling them as they are labelled in Figure 6 (e.g. "PA1234-C DBLMSP2").

Thank you for identifying this - we should have added these sequences to our zenodo release, in addition to the raw inputs and workflows, because the computational pipeline to regenerate our sequences is indeed demanding. We have made a new release on zenodo (https://zenodo.org/record/8171279) that includes, in addition to the raw inputs, all sequence files analysed in the paper ('output_analysed_sequences.tar.gz') .

2. Important point: I feel the nomenclature and concept needs clarifying, in particular the concept of "dimorphism". The author insist that these genes have two, and exactly two forms; and then proceed to show a whole variety of different forms, 28 recombination

breakpoints, gene conversions. So these domains are not dimorphic at all in the strict sense of the word, they're highly polymorphic, although each SNP within the domain appear to be dimorphic. It may be more correct to say that, for each locus, ancestries coalesce to exactly two individuals (to simplify, perhaps one could say that there are two major lineages that frequently interact with each other). I think the authors should spend more effort in the intro and discussion to clarify this.

Thank you for highlighting this. We previously focussed too much of the paper narrative on the term ‘dimorphism’ (historically, first used in the late 1980s by Tanabe *et al.* when studying polymorphisms in gene MSP1 (doi.org/10.1016/0022-2836(87)90649-8)). Indeed, while we have two deep splits in each of DBLMSP and DBLMSP2, there are also more than two amino-acids at certain positions as well as extensive recombination: mainly within each lineage but also between lineages. We have thus removed the term ‘dimorphism’ altogether from the paper, except in the discussion (lines 330-332):

‘The existence of exactly two deeply-diverged lineages that have not recombined in specific P. falciparum genes, historically called ‘allelic dimorphism’ in the malaria literature, has been a long-standing puzzle (8,14).’

To clarify the narrative we also start the results with a new Fig. 2 showing only the three main lineages across the two genes, before showing the recombination (Fig. 3) and gene conversion (Fig. 4). We have also removed the HMM logos from the main text (old Fig. 3) as we find it detracts from our new results centred on the evolution of these two genes (see next comment).

3. Important point: Related to the above, one problem with using the DBLMSP and DBLMSP2 gene pair is that you're convolving "dimorphism" with paralogy. I can see that this gene pair was a logical choice in terms of showing the advantages of your pipeline, whose strength is primarily to resolve similar paralog sequences. But it does bring you into this rather confusing space where you're essentially analyzing the two DBs as a single entity (some sort of "pseudo-diploid"), and you're in fact showing that they are actually trimorphic (three lineages in your Figure 6). I think a lot of readers may lose the plot at this point (the story would be a lot simpler had you picked other dimorphic genes). I believe you need to think how to guide the reader through these analyses.

Historically, dimorphism referred to orthologous variation only, while, as you pointed out, we end up (after identifying shared sequence, in what is now Fig. 1) studying the DBLMSP and DBLMSP2 paralogs jointly. We indeed have at least a trimorphism altogether. To remove this confusion we have removed the term ‘dimorphism’ entirely, except in the discussion to place our results in the context of historical work on other

surface antigens. We also now introduce our tree with three lineages earlier, in Fig. 2, shown below. We also clearly state that seeing three forms across the two genes is unexpected given both genes have (at least) two deeply-split lineages, which should amount to four lineages in total (lines 190-191).

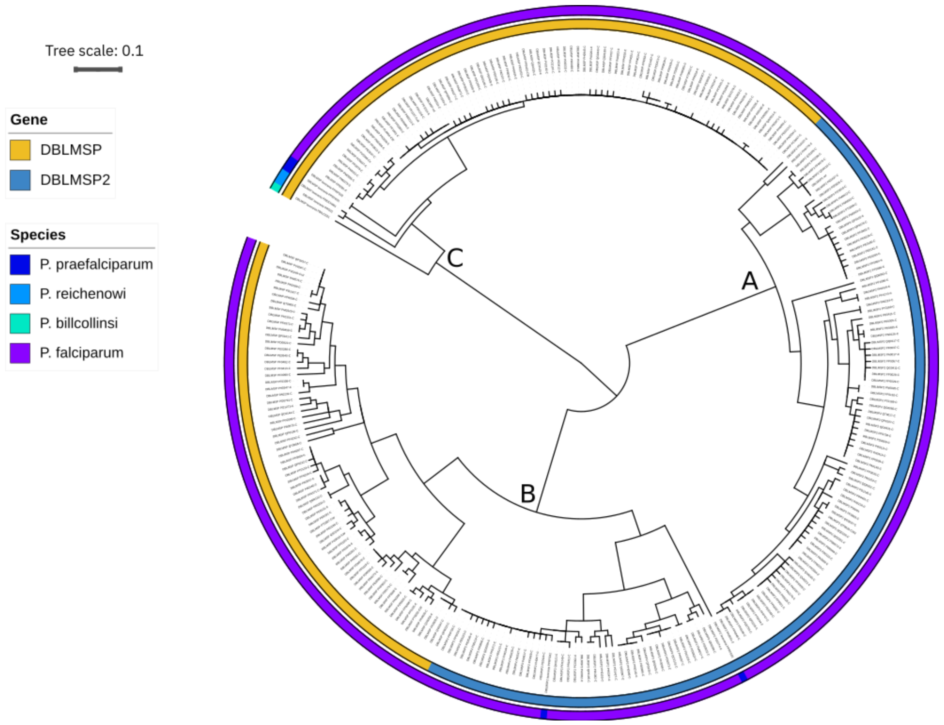

New Fig.2: Deeply-diverged private and shared lineages in DBLMSP1/2. A hierarchical clustering tree of all unique DBL-spanning protein sequences was built. The inner ring colours sequences by gene of origin (DBLMSP, DBLMSP2), and the outer ring shows species of origin, for *P. falciparum* and its three most closely-related species. Three main lineages exist in the tree, labelled A, B and C: lineages A and C contain only representatives of DBLMSP2 and DBLMSP respectively ('private lineages'), and lineage B contains representatives of both ('shared lineage').

We also clarified and strengthened our argument on how our identified gene conversion has created new sub-lineages in the tree: see results section 4 of the revised paper, and new Fig. 5 reproduced below.

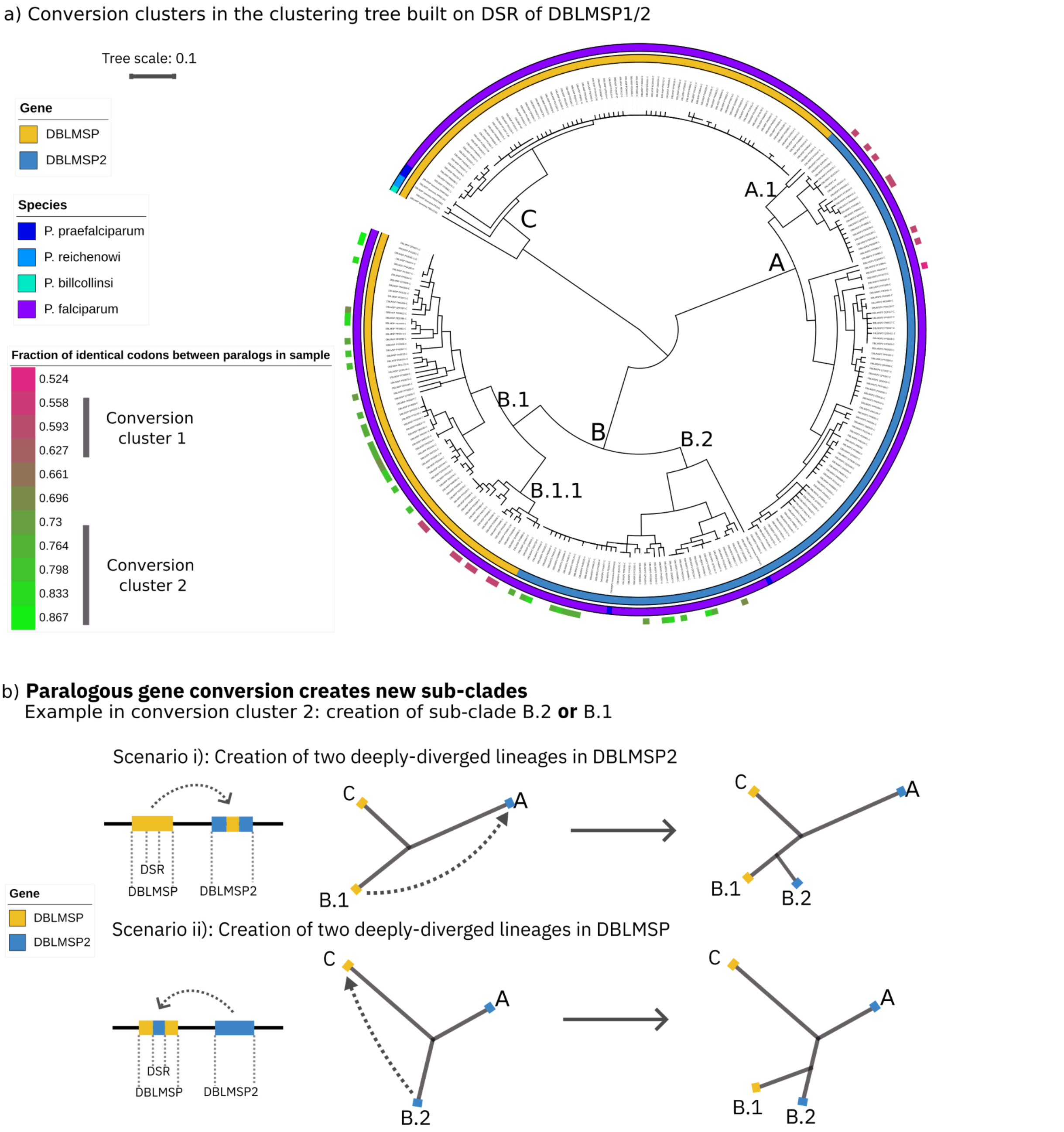

New Fig. 5: Sub-lineage birth by gene conversion between DBLMSP and DBLMSP2. a) The same clustering tree as in Fig. 3 is shown (built in the DBL-spanning region (DSR)), with the addition of an outer ring showing the fraction of identical codons between DBLMSP and DBLMSP2 in sequences where it is > 0.5. These are the sequences inferred to have undergone non-allelic paralogous gene conversion, as shown in Fig. 5. The sequences fall into two clusters (as also shown in Fig. 5) with different fraction identity values, around 0.5-0.6 (conversion cluster 1, pink) and 0.8 (conversion cluster 2, green). b) A simplified schematic showing how gene conversion created two deeply-diverged lineages in either DBLMSP2 (scenario i), or DBLMSP (scenario ii), depending on the direction of the gene conversion.

4. Important point: The proposed model in the discussion is speculative (albeit plausible) and needs to be clearly marked as such. I am afraid that this also makes the manuscript title inappropriate. Even if the model is correct, and an additional form was generated by gene conversion, there is no explanation of how this form persists at ~50% prevalence, so to say that the conversion "drives" dimorphism does not seem correct. Also- the paper does not say whether the duplication leading to the paralogs occurred before or after the species jump. Is there evidence of this paralogy in gorillas?

Firstly, we have added sequences from gorillas and chimpanzees - thank you for this suggestion! The sequences we could reconstruct are in our new Figures 2 and 5 (shown above). We clearly find that the duplication leading to the paralogs precedes the *P. falciparum* - *P. reichenowi* split and thus also the jump from gorilla to humans (lines 174-175). We also use the sequences in other *Plasmodium* to infer a putative direction of gene conversion (lines 288-290), and a possible human-specific evolutionary constraint in DBLMSP2 (discussion: lines 370-376).

In light of this, and in complete agreement that 'drive' is too strong a word, we updated the paper title to 'Evolution of deeply-diverged lineages in two paralogous cell-surface antigens of the malaria parasite *P. falciparum*'.

Reviewer #2:

Letcher et al have undertaken a study in which they have employed a variant detection pipeline, gramtools, which uses genome graphs to assess variants that are called with a range of different genotype callers and is less affected by divergent haplotypes than other callers. After extensive validation of this pipeline they have applied it merozoite surface proteins in P. falciparum which appear to show highly diverged haplotypes, and are subject to frequent ectopic recombination.

The improved variant calls suggest haplotypes are shared between the two MSPs, and further analysis of recombination patterns and phylogenetics strongly suggest this is a case of gene conversion.

The work is extremely thorough, comprehensive and convincing, the results are notable in the context of malaria evolution. I have little hesitation in recommending it for publication.

Thank you for your positive review!

If I have a criticism to make, it is that the validation of gramtools in the content of Pf takes up a large part of the paper and does not show significant novelty beyond Letcher 21 / Hunt 22. This detracts from the more interesting story of gene conversion between MSPs and the paper might be better served by placing this in the supplementary.

Thank you for this. We agree that the core of the paper is indeed about the evolution of the sequences we obtained from our pipeline, and thus that the paper is best served by reducing the technical details of how we obtained them. To address this we have significantly shortened section 1 of the main results: old Fig. 1 has been moved to the supplementary, and the section was reworded to emphasise only i) that we developed a new pipeline, designed to address GATK's shortcomings in highly-variable genes ii) that we designed a thorough evaluation pipeline (now no longer giving any details) that produced many more confidently-resolved sequences compared to malariaGEN's GATK-based pipeline (section 1: lines 96-111).

In the same vein we also moved the methods section describing the tools in our new pipeline (and rationale for their inclusion) to the supplementary.

Thank you for your thoroughness in comparing this work with our previous work (Letcher 21 / Hunt 22): there is indeed overlap. We would like to nonetheless emphasise that this pipeline does show significant novelty. Letcher 21 presents gramtools and uses gramtools for joint genotyping, while Hunt 22 uses gramtools for adjudication (combining multiple genotypers). In this pipeline, we use gramtools both for adjudication and joint genotyping, combine different tools than in Hunt 22 (Octopus instead of samtools), and added a new method that uses local assembly to reconstruct sequences missing from the genome graph (gapfiller). To clarify these differences, we added this sentence to the text in the supplementary:

'*Compared to our previous work (4,6), we added the tool Octopus (3) prior to adjudication and GapFiller (5) after adjudication, to resolve remaining diverged alleles.*'

Ultimately, though, we totally agree that focussing the paper on analysis of the gene sequences themselves makes the paper more engaging and accessible.

This may leave space for other evolutionary implications of this result (assuming they are not being dealt with elsewhere) such as the biological effects of these haplotypes and whether metrics such as KaKs etc indicate differing selective pressures on both private and shared haplotypes.

Thank you for these suggestions. We note that two of the authors of this study have previously taken part in a study on *in vitro* immunoglobulin binding properties of (a small subset of) DBLMSP/DBLMSP2 haplotypes (https://pubmed.ncbi.nlm.nih.gov/27226583/). Surprisingly, that study found no immunological differences between the tested haplotypes (this is stated in this paper, lines 382-383). It would be interesting to test our new haplotypes in this way. We also think that more human protein targets need to be tested (not just immunoglobulins), and ideally *in vivo* and not *in vitro* (discussion: lines 386-391). This could be the subject of a future biological study, but is beyond the scope of this one.

As to your second suggestion, because of gene conversion, the Ka/Ks of different lineages will not be independent, and so we decided not to explore this.

Instead, we considered further evolutionary implications of our results by adding new sequences from other *Plasmodium* species to our analysis (as suggested by both reviewers 1 and 3). Using these data we found i) that the DBLMSP1/2 duplication predates the jump to humans (lines 174-176) ii) the putatively ancestral DBLMSP and DBLMSP2 sequences from *P. praefalciparum* (new Fig. 2) iii) the possible direction of gene conversion (lines 288-293) and iv) a possible human-specific functional constraint of the DBL domain in DBLMSP2 (lines 370-376). We hope you also find that this new analysis provides interesting evolutionary context and insights to this paper.

General nitpicking below:

L113 - the range of tools appears to be cortex + octopus; these tools (and the reasons for choosing each) should be included

(For the reasons above) We no longer discuss the specific tools of our new pipeline in the main results. We now discuss all the tools in one coherent section of the supplementary, including the rationale for choosing each tool (Table 1 in section ‘Pipeline components’; note this was previously in the methods section).

L127 / supplemental
Numbering and ordering of supplemental figures is either out of order or very confusing.

Thank you, we wanted to label figures by section of the paper they referred to (D for discussion, 1.2 for results section 1..) but this was confusing - they’ve all been relabelled numerically.

L181 / fig s-2.1: without context it is hard to know how high this is - how does it compare to other paralogues that are not involved in cytoadhesion?
To our knowledge, this has not been done in other paralogs because these are hard to genotype properly using short-read data - as shown in this paper. Assuming a single 'seed' event generating shared sequences by gene conversion, however, these numbers are high, as they suggest the shared sequences rose to, and became maintained at, a high frequency from that point.

L256: Analysis appears sound, but it took me a few reads to figure it out, so perhaps some clarification that this is an alignment between genes is needed.

Thank you for highlighting this, we agree that this concept was not sufficiently clear. We thus augmented old Fig. 5 with an explanatory panel a (in what is now Fig. 4), together with a clear figure legend stating what each row refers to:

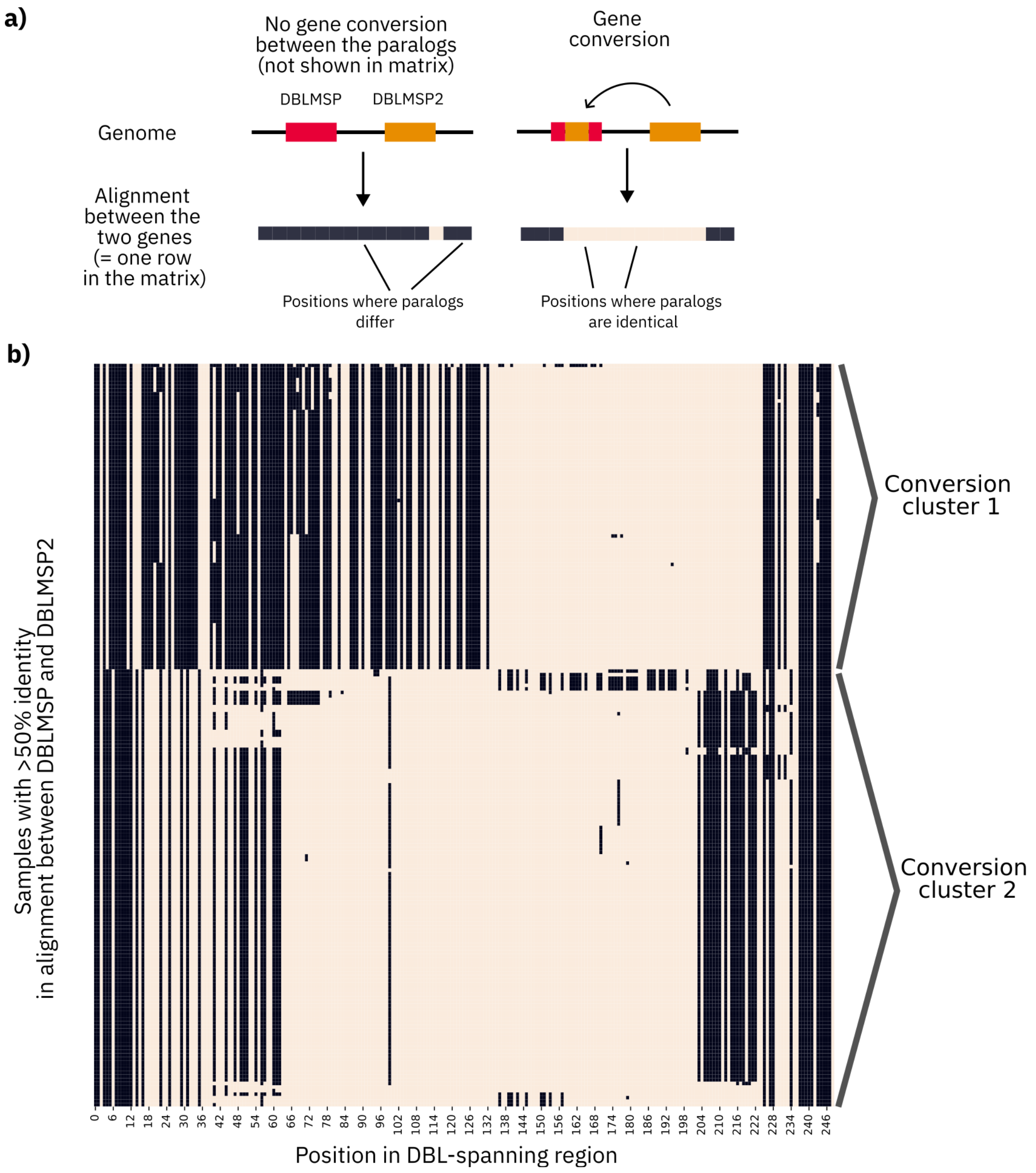

Figure 4. **Evidence for non-allelic gene conversion between DBLMSP and DBLMSP2 in the DSR.** a) This scheme explains the matrix that follows in panel b. For each of the samples in which both DBLMSP1/2 gene sequences were confidently-resolved, we aligned their DNA sequences in the DSR and recorded positions where codons were identical between the two genes (beige cells), versus different (black cells). Gene conversion should appear as contiguous strips of beige cells. b) The 209 samples with > 0.5 identical codons between DBLMSP and DBLMSP2 are shown (rows) at each position of the DSR (columns). The strips of near-all beige indicate likely sequence copying between the two genes in a sample, supporting gene conversion occurring within each genome. Two main clusters of samples can be distinguished visually (labelled cluster 1 and 2 on the right), likely corresponding to two distinct conversion events.

L261 / Fig 5 do branch lengths derive from both the non-converted and converted regions? i.e. is there divergence between samples where gene conversion has occurred between DBs and if so how much?

In our new Fig. 4 we removed the clustering tree on the left as its only point was to highlight the presence of two different conversion clusters (this is now shown on the right-hand side of the matrix). To answer your question, the branch lengths come from the DBL-spanning region (all positions on the x-axis), and so does include some non-converted regions (black columns in the matrix).

Inside the converted regions, there is some sequence divergence. Notably, at some positions that were gene-converted in both conversion clusters, the amino acids differ. This is shown in supp. Fig. 20 (e.g., amino acids in the bottom-left of that figure), and referred to in the main text on lines 265-267:

‘Two main conversion clusters can be visually distinguished in Figure 4 panel b, with different breakpoints (start and end positions of the beige strips); these are also different at the sequence level (supp. fig. 20).’

Either the gene-converted amino-acids were initially different, or they were the same and the two conversion clusters subsequently diverged - we don’t really know which.

L280: This figure is *very* complex and I'm not sure it adds much to the plot.

Thank you, we agree with your point and have removed this figure. Instead, we now show a much simplified version of this Figure, as Fig. 2:

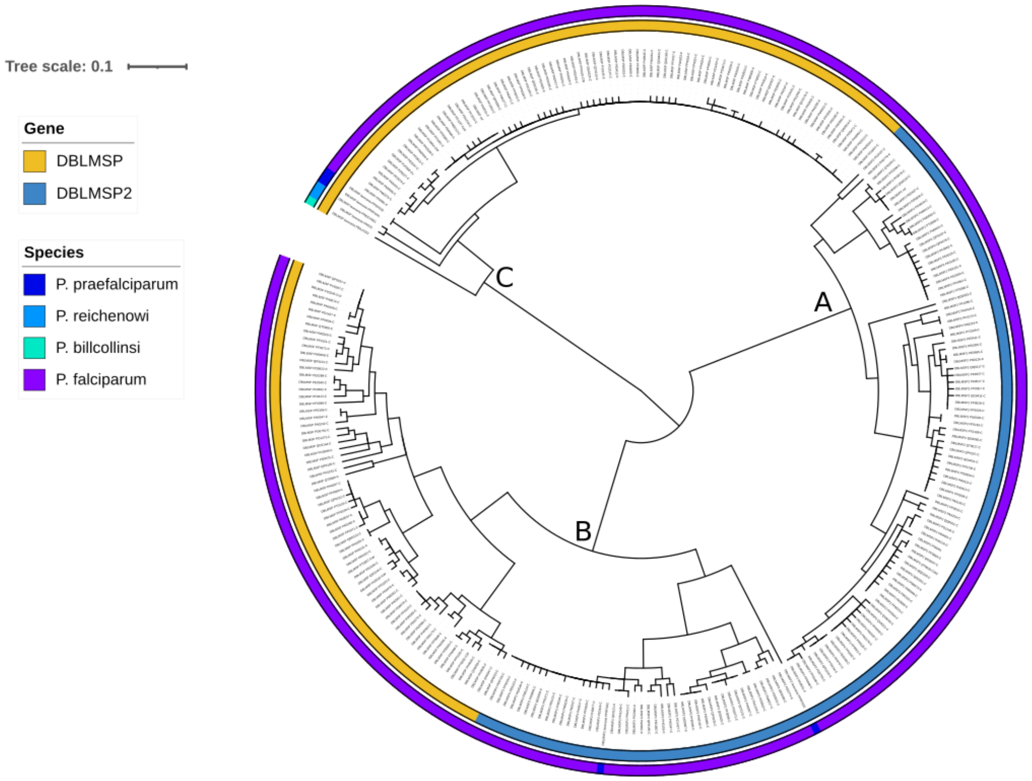

New Fig.2: Deeply-diverged private and shared lineages in DBLMSP1/2. A hierarchical clustering tree of all unique DBL-spanning protein sequences was built. The inner ring colours sequences by gene of origin (DBLMSP, DBLMSP2), and the outer ring shows species of origin, for *P. falciparum* and its three most closely-related species. Three main lineages exist in the tree, labelled A, B and C: lineages A and C contain only representatives of DBLMSP2 and DBLMSP respectively ('private lineages'), and lineage B contains representatives of both ('shared lineage').

Notice this Figure also includes the sequences from the three most closely-related ape-infecting *Plasmodium* species to *P. falciparum*. This Figure now occurs before showing the recombination (Fig. 3) and gene conversion (Fig. 4) results, to provide a global picture first. We then show how gene conversion can create two deeply-diverged lineages in a new Fig. 5:

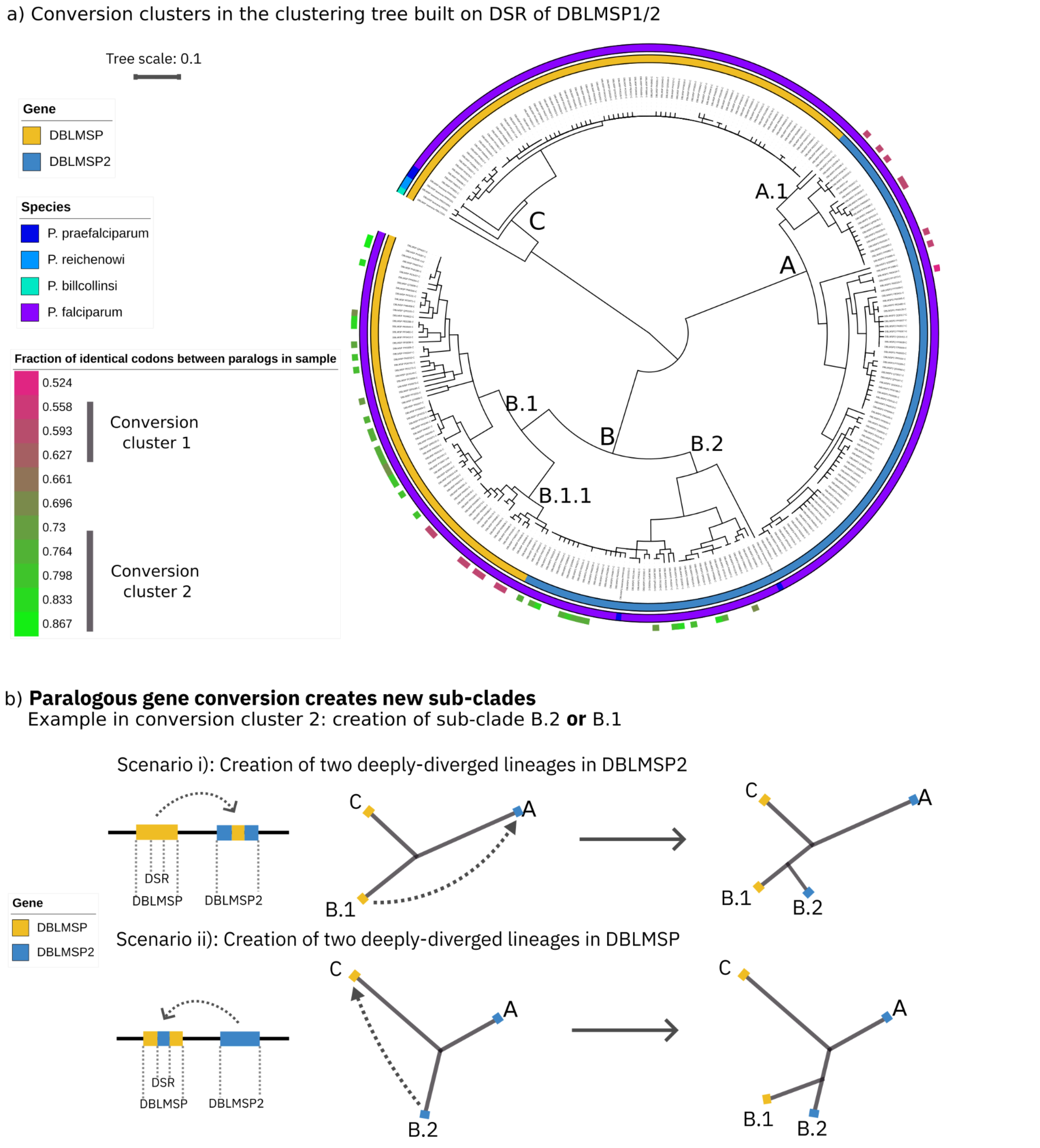

New Fig. 5: Sub-lineage birth by gene conversion between DBLMSP and DBLMSP2. a) The same clustering tree as in Fig. 3 is shown (built in the DBL-spanning region (DSR)), with the addition of an outer ring showing the fraction of identical codons between DBLMSP and DBLMSP2 in sequences where it is > 0.5. These are the sequences inferred to have undergone non-allelic paralogous gene conversion, as shown in Fig. 5. The sequences fall into two clusters (as also shown in Fig. 5) with different fraction identity values, around 0.5-0.6 (conversion cluster 1, pink) and 0.8 (conversion cluster 2, green). b) A simplified schematic showing how gene conversion created two deeply-diverged lineages in either DBLMSP2 (scenario i), or DBLMSP (scenario ii), depending on the direction of the gene conversion.

To get the full context of these new figures, please see the revised main text.

L318: This section feels like a response to a reviewer question, but IMO doesn't add a lot to the paper.

This section indeed does not prove or disprove anything: we did not detect gene conversion from one generation to the next in available data, but this does not mean it has never happened in the wild. We still find this (small) section important as it shows i) how different types of data (repeated evolution, genetic crosses) can be used to directly test our indirect observation of gene conversion and ii) that such data have indeed revealed gene conversion in *P. falciparum* before, in a different pair of genes (lines 325-327). However, we removed the paragraph introducing this section in section 4, as it is not central to the paper's story.

Reviewer #3:

The authors have analyzed sequence reads from the MalariaGEN project (Plasmodium falciparum genome sequences from many thousands of samples). They have used a new pipeline they devised (and published in Ref.28) to characterize previously overlooked divergent allele sequences of two surface antigens (DBLMSP and DBLMSP2). I would not claim to fully understand the pipeline that leads to improved resolution of these sequences, but I think that they present convincing evidence of the efficacy of this method (Fig. 2). They then go on to show that there is evidence of recombination (probable gene conversion) between divergent sequences, within and between the two nearby loci. I found this very interesting.

Thank you!

Major comments

1. Given that it is already known that these two loci are dimorphic, would a standard assembly approach, run twice (using the two forms of each locus as reference) work just as well to resolve these sequences?

Thank you, this is an interesting question. We think it would partially work, but that our genome graph approach is much more sensitive and comprehensive. Here are some reasons why. Firstly, given the gene conversion we found between these two paralogs (Fig. 4), a single-reference based approach is likely to map ‘converted’ reads preferentially in one gene (the one containing the shared haplotype in whichever reference), while in our genome graph, all haplotypes are simultaneously available, and

reads can more easily map to both. Secondly, we showed that there is in fact a lot of recombination between haplotypes (Fig. 3) - such recombinants are in general much harder to reconstruct on a linear reference. Thirdly, there is the question of reconciliation: which gene sequence should one pick once it has been reconstructed in two references? This question is in fact directly answered in the genome graph framework.

In summary we have not tried the approach you suggest, though it may partially work. We argue that using a dedicated framework with 1,000s of available haplotypes embedded in a graph, rather than just two and on distinct references, is better adapted to finding novel haplotypes.

2. The authors note that Plasmodium falciparum arose by transmission of a gorilla parasite via a tight bottleneck, and discuss a model (Fig. 7) for the subsequent evolution of dimorphic loci through gene conversion between paralogous loci. So I was surprised that they did not include the DBLMSP and DBLMSP2 sequences from the gorilla parasite in their analysis; Otto et al. (2018; Ref.17) presented three genome sequences from this species.

Thank you for this highly valuable suggestion. We have reconstructed sequences from other *Plasmodium* species using both the long-read based assemblies and short-read sequencing data in Otto *et al.* (2018) (for how, please see the new methods section in lines 498-530).

The sequences we could reconstruct are in new Figures 2 and 5 (please see responses above for these figures). We clearly find that the duplication leading to the paralogs precedes the *P. falciparum* - *P. reichenowi* split and thus the species jump from gorilla (section 2: lines 174-175). The addition of gorilla sequences was also informative as to the putative direction of gene conversion (section 2: lines 288-290), and a possible human-specific evolutionary constraint in DBLMSP2 (discussion: lines 370-376).

This new addition also led us to improve our reasoning of how our identified gene conversion has created new sub-lineages in the tree, leading us to remove Fig. 7 and replace it with Fig. 5 panel b.

Minor comments

1. Line 32, and later: I think the authors should avoid using "Pf" to denote P.falciparum. Similarly, line 98 and later, I think the authors should avoid using "DBs". Finally, lne 144 and later, I think the authors should avoid using "MSA". Filling the paper with

acronyms/abbreviations does not help its readability, and shortens the paper only marginally.

Thank you, we agree. We replaced ‘DBs’ with ‘DBLMSP1/2’ throughout the paper, which is more explicit. We also replaced ‘Pf’ with *P. falciparum* and MSA with ‘multiple-sequence alignment’.

2. Figure 1. I did not find this helpful - I found it lacked sufficient detail/information to clarify anything beyond what is already stated in the text.

Our intention with this figure was to allow a ‘quick’ overview of the features of our new pipeline. We also argue that understanding the difference between adjudication (combining multiple different genotypers) and joint genotyping, and understanding the order of the different steps, are helped by a visual representation.

However, through this revision (and as also suggested by R2) we have found that the paper is best served by moving the technical details of the pipeline and its evaluation to the supplementary. We thus moved Fig. 1 and the text/methods related to details of the pipeline to the supplementary - we hope you agree this helps focus on the analysis of DBLMSP1/2 sequences and their evolution.

3. Figure 2a and line 360: With two amino acids at a site in different alleles, the heterozygosity can rise to a maximum value of 0.5, when the two are at equal frequencies. In Figure 2a (right panels), for both genes there are numerous sites with values greater than 0.5, seemingly indicating that there are more than two alleles. Is this due to a number of rare amino acids at these sites?

Yes, you are correct, at these positions there are more than two amino acids. As shown in Fig. 3 of our previous submission, about two amino acids are present at high frequency at each position (three for some parts), but old supplementary Figure 2.2 (now supp. fig. 14) shows the total number of amino acids at each position, which can reach up to 6 or 8. Note that we have moved old Figure 3 to the supplementary, as we focussed our revision on the evolution of the deeply-diverged lineages instead (with new Figures 2 and 5).

4. Line 179, and lines 558 onwards: could clarify whether overlapping 10-mers are considered. (For example, if the 10-mer at sites 101-110 is considered, are those at 102-111, 103-112, etc., also considered?)

Thank you for this, yes overlapping positions are considered, this was added to the text defining private/shared peptides:

'To define sequence sharing, we broke each sequence in our multiple-sequence alignment of all confidently-resolved DBLMSP1/2 sequences into overlapping peptides of length 10 [...]'

This definition has been moved from the methods to the supplementary, because in our revision we use a new Fig. 2 to more simply define shared and private lineages on a tree - not based on 10-mers. This makes for easier reading while maintaining the point. In the supplementary, we still use the 10-mer based definition to show that these definitions are essentially equivalent (supp. fig. 11) and to show private/shared peptide frequencies (supp. fig. 12).

5. Figure 3. I did not find the explanation at lines 207-209 clear. Perhaps giving examples would help. Am I correct to think that the motif LRWFREWST, found in both genes near the bottom right, is a counterexample to what is stated in lines 207-209?

We agree that Fig. 3 of our submission is difficult to understand in detail.
For example, for 'LRWFREWST': it is of size 9, and because sharing was defined on 10-mers and the amino acid just before that motif is different in the two genes ('Y' vs 'F'), that region is classified as private, despite those 9 amino acids being identical. It is indeed a counterexample to the statement at lines 207-209; we have thus amended the legend as: 'In-between diverged N- and C-terminal regions, there is mostly [...]'.

We have moved Fig. 3 to the supplementary (supp. fig. 13) because our main point was that there are three main lineages, two private, and one shared - this point is much more clearly illustrated in our new Fig. 2.

6. Line 285: Please give the number of unique protein sequences at this point.

Thank you, this number was added: 278 sequences in total (line 150). Note that 7 of these are now from other *Plasmodium* species, as per your major comment above.

7. Fig.6: I assume that the "subform" mentioned at line 314 is clade A.1.

Yes; to clarify this, clade A.1 is now mentioned explicitly in the text (line 295), and in reference to new Fig. 5 (in replacement of old Fig. 6, that we removed as it was overly complicated - as suggested by R2).

8. Line 443: explain MOI.

Thank you, we now state on lines 408-409:
‘However, samples with multiple co-occurring strains (multiplicity of infection (MOI) > 1) are common in P. falciparum’

9. Line 461: 240kbp ? - which 240kbp ?

We’ve modified the text as follows, to state that the 240kbp were taken evenly from the *P. falciparum* core genome (we hope this is sufficient for you, please let us know if you’d like us to also provide a bed file with the regions - we personally do not view this as crucial) (lines 426-429):
‘We measured these in alignments falling inside P. falciparum ‘core genome’ as defined by (33) by excluding highly repeated or variable genomic regions like the telomeres and var genes. We selected a 240kbp subset of this core genome, spread evenly across all 14 P. falciparum chromosomes.’

10. Line 463: "lied" should be "lay"

Thank you, done

11. The numbering of the supplemental figures is odd.

Thank you, we wanted to label figures by section of the paper they referred to (D for discussion, 1.2 for results section 1..) but this was confusing - they’ve all been relabelled numerically.

Editorial Assessment

Dear Dr Letcher,

Thank you for your patience while your manuscript "Gene conversion drives allelic dimorphism in two paralogous surface antigens of the malaria parasite P. falciparum" was peer-reviewed at PLOS Biology. It has now been evaluated by the PLOS Biology editors, an Academic Editor with relevant expertise, and by three independent reviewers.

You'll see that reviewer #1 is positive, but worries about availability of the sequence assemblies (please ensure that you are fully compliant with the PLOS data availability policy!),

This is now done, and we are very grateful that reviewer #1 and the editor picked up on this. While the sequences were re-generatable using the initially provided software and code, this is computationally costly and the sequences are an important deliverable; it is thus crucial to include them directly. Apologies for missing this in the previous round.

has semantic issues with the word “dimorphism” (and its relationship to paralogy – this also confused me first time round)

We have thought carefully and reworded the whole paper: we removed the term “dimorphism” entirely (except for a mention in the Discussion), and now talk of depth and number of lineages instead. This is much more comprehensible to a wider biological audience.

. , and wants you to mark the mechanistic model as speculative and tone down the title.

Done: our proposed new title is ‘Evolution of deeply-diverged lineages in two paralogous cell-surface antigens of the malaria parasite *P. falciparum*’, and we have replaced Fig. 7 (illustrating the model) with panel b of Fig. 5, that is much more explicit. Further, having reconstructed sequences from other *Laverania* enabled new insights and statements, including that the duplication leading to the paralogs preceded the *P. falciparum* - *P. reichenowi* split and thus also the jump from gorilla to humans (section 2: lines 174-175), and the putative direction of gene conversion (section 2: lines 288-290)

Reviewer #2 is also positive, but thinks that validation of the pipeline duplicates somewhat your previous paper and detracts from the main message (s/he suggests moving it to the supplement, leaving space for considering other stuff).

We agree with the stated overlap (though see our response to R2 for how our pipeline does show significant novelty - we have modified the text describing our new pipeline to clarify this). We also agree that the technical details provided in section 1 were very bioinformatics-oriented, with a risk of reduced readership and detracting from the more interesting sequence-based and evolutionary analysis of these two genes. We have thus moved old Fig. 1 and the technical details of the pipeline and its evaluation to the supplementary. The new section 1 text now simply states that we developed a new pipeline, the reasons why, and that we obtained many more confidently-resolved sequences than previously possible (section 1: lines 96-111), deferring the details to the supplementary.

Reviewer #3 is similarly positive, but wonders if you could have obtained the result simply by using the two haplotypes as two parallel reference samples,

This is addressed above

and asks about the timing with respect to the zoonotic jump from gorillas.

Done! (see comments above)

The Academic Editor, in discussing these comments, said "R1 and R3 query the gorilla orthologs and R3 specifically asks for these to be included in the analysis which doesn't seem unreasonable in light of the authors' bottleneck hypothesis. I think this additional analysis would justify 'major revision'.

This is why we love peer review, a most helpful suggestion - this is now done.

R1 emphasises the usefulness of the method details but R2 wants them shifted to suppl. I think if the method has been published separately as R2 claims then the suppl is appropriate.

As stated above, this is a new pipeline that uses gramtools (which has been published before) but also adds new components. However, we completely agree with focussing the paper on the analysis and evolution of our newly-resolved DBLMSP1/2 sequences, to access a broader audience; we thus moved the technical/bioinformatic details on the pipeline and its evaluation to the supplementary (see above).

I think providing the assemblies as requested by R1 is also appropriate."

100% agreed and done (see updated Data Availability section).

In light of the reviews, which you will find at the end of this email, we would like to invite you to revise the work to thoroughly address the reviewers' reports.

Given the extent of revision needed, we cannot make a decision about publication until we have seen the revised manuscript and your response to the reviewers' comments. Your revised manuscript is likely to be sent for further evaluation by all or a subset of the reviewers.

We expect to receive your revised manuscript within 3 months. Please email us (plosbiology@plos.org) if you have any questions or concerns, or would like to request an extension.

At this stage, your manuscript remains formally under active consideration at our journal; please notify us by email if you do not intend to submit a revision so that we may withdraw it.

Thank you again for your submission to our journal. We hope that our editorial process has been constructive thus far, and we welcome your feedback at any time. Please don't hesitate to contact us if you have any questions or comments.

Sincerely,

Roli Roberts

Roland Roberts, PhD
Senior Editor
PLOS Biology
rroberts@plos.org

Guidelines for resubmission

IMPORTANT - SUBMITTING YOUR REVISION

Your revisions should address the specific points made by each reviewer. Please submit the following files along with your revised manuscript:

1. A 'Response to Reviewers' file - this should detail your responses to the editorial requests, present a point-by-point response to all of the reviewers' comments, and indicate the changes made to the manuscript.
*NOTE: In your point-by-point response to the reviewers, please provide the full context of each review. Do not selectively quote paragraphs or sentences to reply to. The entire set of reviewer comments should be present in full and each specific point should be responded to individually, point by point.
You should also cite any additional relevant literature that has been published since the original submission and mention any additional citations in your response.

2. In addition to a clean copy of the manuscript, please also upload a 'track-changes' version of your manuscript that specifies the edits made. This should be uploaded as a "Revised Article with Changes Highlighted" file type.

Re-submission Checklist
When you are ready to resubmit your revised manuscript, please refer to this re-submission checklist: https://plos.io/Biology_Checklist

To submit a revised version of your manuscript, please go to https://www.editorialmanager.com/pbiology/ and log in as an Author. Click the link labelled 'Submissions Needing Revision' where you will find your submission record.

Please make sure to read the following important policies and guidelines while preparing your revision:

Published Peer Review
Please note while forming your response, if your article is accepted, you may have the opportunity to make the peer review history publicly available. The record will include editor decision letters (with reviews) and your responses to reviewer comments. If eligible, we will contact you to opt in or out. Please see here for more details: https://blogs.plos.org/plos/2019/05/plos-journals-now-open-for-published-peer-review/

PLOS Data Policy
Please note that as a condition of publication PLOS' data policy (http://journals.plos.org/plosbiology/s/data-availability) requires that you make available all data used to draw the conclusions arrived at in your manuscript. If you have not already done so, you must include any data used in your manuscript either in appropriate repositories, within the body of the manuscript, or as supporting information (N.B. this includes any numerical values that were used to generate graphs, histograms etc.). For an example see here: http://www.plosbiology.org/article/info%3Adoi%2F10.1371%2Fjournal.pbio.1001908#s5

Blot and Gel Data Policy
We require the original, uncropped and minimally adjusted images supporting all blot and gel results reported in an article's figures or Supporting Information files. We will require these files before a manuscript can be accepted so please prepare them now, if you have not already uploaded them. Please carefully read our guidelines for how to prepare and upload this data: https://journals.plos.org/plosbiology/s/figures#loc-blot-and-gel-reporting-requirements

Protocols deposition
To enhance the reproducibility of your results, we recommend that if applicable you deposit your laboratory protocols in protocols.io, where a protocol can be assigned its own identifier (DOI) such that it can be cited independently in the future. Additionally, PLOS ONE offers an option for publishing peer-reviewed Lab Protocol articles, which describe protocols hosted on protocols.io. Read more information on sharing protocols at
https://plos.org/protocols?utm_medium=editorial-email&utm_source=authorletters&utm_campaign=protocols
