# Supplementary material for: Role for gene conversion in the evolution of cell-surface antigens of the malaria parasite Plasmodium falciparum
Source: PLoS Biol. 2024 Mar 7;22(3):e3002507. doi: 10.1371/journal.pbio.3002507 (PMC10919680; doi:10.1371/journal.pbio.3002507)
Supplement: Attachment — Submitted filename: round_2_revision_response_to_reviewers.pdf [file pbio.3002507.s032.pdf]

Dear Editor,

In this revision, we have addressed the following points you have raised:

- We agree with changing the title to be more appealing, and, taking into account your suggestions, would like to propose the following new title: ‘Role for gene conversion in the evolution of cell-surface antigens of the malaria parasite *P. falciparum*’
- We have implemented full compliance with the PLOS data policy by releasing, on zenodo, a set of data files enabling reproducing all main and supplementary figures, together with instructions for what pieces of code to run (the code being released on zenodo as well): https://zenodo.org/records/10451641
- We were missing references to two computational tools we used (Snakemake and Singularity), and added them to the main text

We further addressed reviewer #3’s second round of review, for which we are grateful and find has again improved the paper. The main points of our revision are summarised below:

- We have modified Fig. 2 to make the species of origin of each sample more visible
- We no longer refer to ‘conversion clusters’, a term first introduced in Fig. 4 that led to confusion when we started discussing gene conversion in the context of the tree in Fig. 5. We now use ‘conversion events’ throughout the paper instead.
- Fig. 5 has been simplified considerably, removing extraneous data and adding annotation to help the reader. We no longer show values in the outermost ring of Fig. 5 panel a, and simply label samples as belonging to conversion events 1 or 2 (having defined these in Fig. 4). We also added stars to Fig. 5 panel a labelling which sub-clades of the tree were born from the two gene conversion events, and discuss this in the main text
- We simplified our argument on the direction of sequence pasting for gene conversion event 1.
- We have added three figures to the supplementary in support of answering reviewer #3’s comments - these are referred to in the main text and are also reproducible as per above

Please find our detailed response to reviewer #3 below.

Reviewer #1:

I have now reviewed the authors' replies and I am happy to accept the manuscript for publication.

Reviewer #3:

I have the revised version, and find it greatly improved. I have only a few comments, which you will find below.

We would like to thank reviewer #3 for engaging so thoroughly with our new version; their comments were once more highly valuable and we feel addressing them has further improved the paper!

PBIOLOGY-D-23-00695R2

LETCHER et al.

Line 169: DBLMSP2 was not found in P. billcollinsi, even though an assembled genome is available. Laverania retain strong synteny, so it should be possible to comment whether the gene is indeed missing. Do the more divergent Laverania species have DBLMSP2? Or was it created by a duplication event after the divergence of the ancestor of P. billcollinsi?

Thank you for highlighting this. The more divergent *Laverania* species indeed do not have DBLMSP2: a full-length DBLMSP is only present in *P. praefalciparum*, *P. reichenowi* and *P. billcollinsi* and not in more diverged species, and a full-length DBLMSP2 is only present in *P. praefalciparum* and *P. reichenowi*. Our data are thus consistent with DBLMSP2's origin dating to between the *P. falciparum-P. billcollinsi and P. falciparum-P. reichenowi*. We have updated the main text to reflect this:

'*To probe the evolution of DBLMSP1/2, we searched for orthologs in the six closest known relatives of P. falciparum (all part of sub-genus Laverania). Using sequencing data and genome assemblies from Otto et al. (17) we could reconstitute up to 2 sequences per species (see methods). For DBLMSP, we found clear orthologs in the three most closely-related species to P. falciparum: P. praefalciparum, P. reichenowi and P. billcollinsi (in increasing order of divergence time from P. falciparum), but not in the more diverged species. For DBLMSP2, we found a clear ortholog in P. praefalciparum and P. reichenowi only. We thus date the ancestral duplication of DBLMSP to DBLMSP2 to between the P. falciparum-P. billcollinsi and P. falciparum-P. reichenowi splits, and thus before the jump of P. falciparum from gorilla to human.*'

To validate this finding, we followed your recommendation to look for additional genes in the Laverania assemblies we have from Otto *et al.* (2018). We found that AMA1, a well-conserved and single-copy gene, and MSP11, a gene located in-between DBLMSP and DBLMSP2, are present in full length in all Laverania assemblies (except *P. blacklocki* for MSP11, but that assembly is low quality according to both us and the Otto *et al.* (the authors) - see new supp. fig. 13 for why). This confirms we are able to recover homologs across all species, and suggests that DBLMSP and DBLMSP2 are truly absent in the Laverania assemblies we could not find them in. We updated the methods as follows (the last sentence is new):

'*To find DBLMSP and DBLMSP2 in the assemblies [...] we mapped the gene sequences from our gramtools-based pipeline to each assembly using minimap2 (58) (preset: '-x asm20'). [...] For P .gaboni we found a small region (~800bp) redundantly matching to both DBLMSP and DBLMSP2 that we discarded, and for P. adleri and P. blacklocki we obtained no hits. To confirm that DBLMSP1/2 were indeed missing in the more distantly-related Laverania, we confirmed we could find orthologs of genes in the immediate vicinity of DBLMSP/DBLMSP2 on the P. falciparum genome, including MSP11 located in-between DBLMSP and DBLMSP2, as well as AMA1, a single-copy and well-conserved gene (supp. fig. 13). For AMA1, correct open-reading frames were obtained using Liftoff (60).*'

We refer the reviewer to our new supplementary figure 13 to visualise these data in full.

Line 176: It would be strange if DBLMSP2 has retained a conserved function only in P. falciparum. That would imply that in both P. reichnowi and P. praefalciparum (and P. billcollinsi, if the duplication happened earlier) the gene was independently inactivated subsequent to divergence from the ancestor with P. falciparum; not very parsimonious to suggest the gene was required all along the specific lineage ;eading to P. falciparum (through various host species), but not in any divergent lineages.

Thank you for raising this. We agree that the scenario you describe is not particularly parsimonious. The problem is that while we have ample data in *P. falciparum*, we only have 2 resolved sequences of DBLMSP and DBLMSP2 each for *P. praefalciparum* and *P. reichenowi*. The data we have in *P. falciparum* are consistent with DBLMSP2 being more highly constrained than DBLMSP (as assessed from the number of genes with premature stop codons); for the other two species, the opposite might hold, but we would need population-level data to assess this (and this does not currently exist). We have therefore updated the paragraph in the discussion to be more cautious:

‘*In terms of evolutionary constraints, we note that in P. falciparum, DBLMSP2 appears more highly-constrained than DBLMSP: of 234 DBLMSP1/2 gene sequences with premature stop codons, 196 lie in DBLMSP and 38 in DBLMSP2. By contrast, in P. reichenowi and P. praefalciparum, all 4 identified DBLMSP orthologs had a complete open-reading frame (2 in each species), while 1 of 4 DBLMSP2 orthologs did (in P. praefalciparum). This raises the intriguing possibility of a human-specific function (or constraint) having evolved in DBLMSP2. To fully test this will require population-level data in P. reichenowi and P. praefalciparum.*’

We now say ‘human-specific function (or constraint)’ to reflect the possibility that complete DBLMSP2 ORFs have been maintained throughout all lineages since its birth by gene duplication, not just in the one leading to *P. falciparum*.

Figure 2: The P. praefalciparum alleles are not easy to see. But having found them, it then seems remarkable that one within the B lineage is nested within a radiation of P. falciparum alleles. How can that happen?

Thank you for highlighting this! Firstly, we agree the Laverania alleles are not easy to spot, so we have updated Figure 2 to increase the width of the coloured circle showing species of origin of each sample:

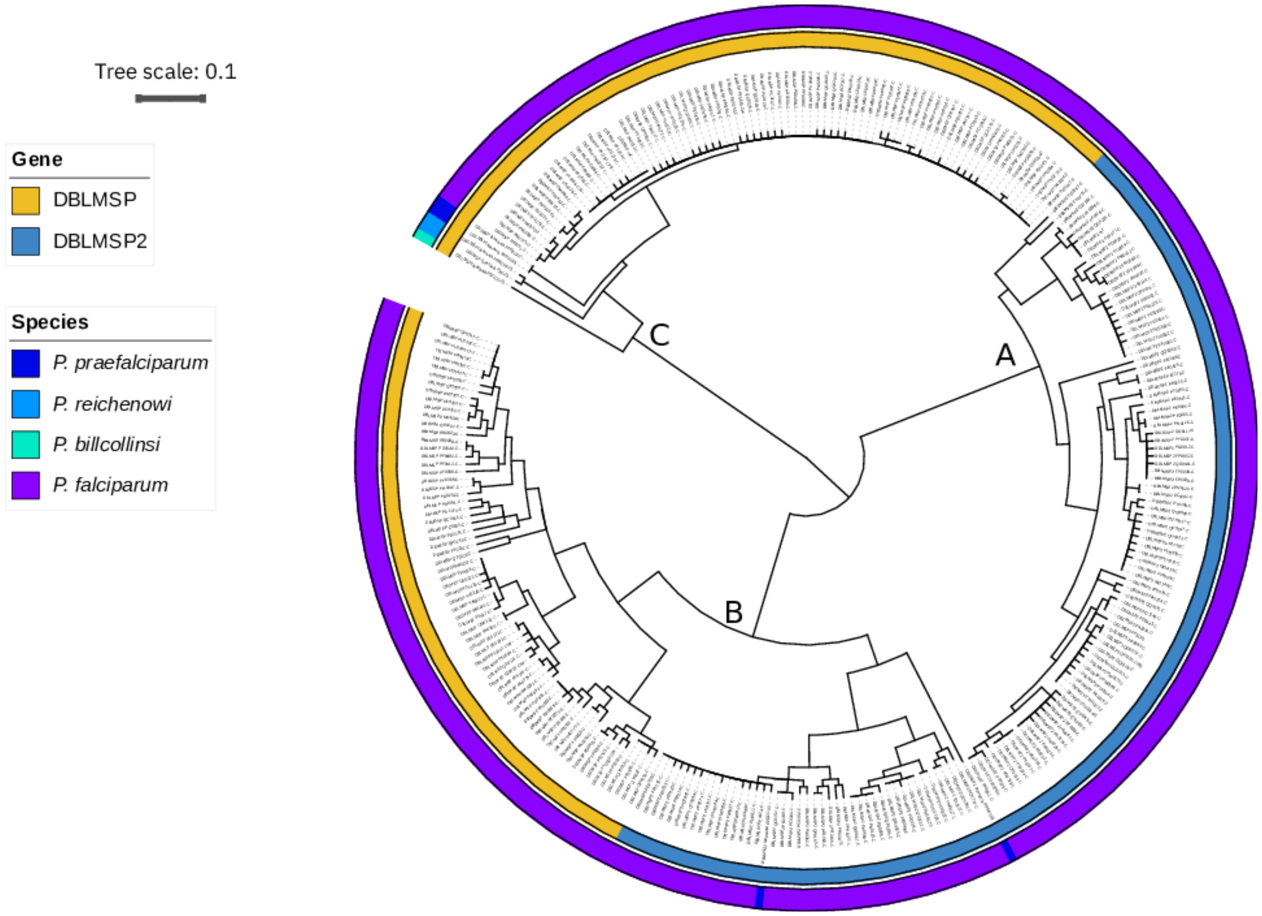

Secondly, we know that *P. praefalciparum* and *P. falciparum* are very close to each other genetically. As stated in the main paper text, *P. falciparum* likely arose very recently by zoonosis from *P. praefalciparum*. Otto *et al.* (2018) date the event to ~50k years ago, and also find that the divergence time of *P. falciparum* to *P. reichenowi* is ~10-fold that to *P. praefalciparum*. It therefore makes sense that *P. praefalciparum* alleles cluster with *P. falciparum* alleles. We indeed found that, in clustering trees built on full-length DBLMSP and DBLMSP2, the *P. praefalciparum* alleles all nested with *P. falciparum* alleles. We also observed this in AMA1, a gene that was found in single-copy in all Laverania assemblies (as per our reply to your comment above). These trees are shown in new supplementary figures 14 and 15.

Thus, overall this ‘allele clustering’ is likely simply a product of *P. falciparum*’s origin from a (very small) set of *P. praefalciparum* individual(s). We now make a note of this in the main text:

‘*The species of origin of each DBLMSP/DBLMSP2 sequence is shown in Fig. 2 (outer ring). For DBLMSP, the orthologs fall in a distinct sub-lineage of lineage C and consistently with the known Laverania phylogeny. For DBLMSP2, the two orthologs in P.*

reichenowi are pseudogenes (consistent with prior knowledge (17)) and not shown in the tree (premature stop codons near the start codon make the DSR incomparable). The two identified orthologs in P. praefalciparum both fall in the shared lineage B: one has a premature stop codon (rightmost P. praefalciparum sample in lineage B), and the other lies nested inside a clade of P. falciparum alleles. We found this is consistent with P. falciparum's recent origin from P. praefalciparum via a tight bottleneck: in trees of full-length DBLMSP and DBLMSP2 sequences and of a single-copy, well-conserved gene (AMA1), P. praefalciparum alleles also nest inside P. falciparum clades (in contrast to alleles from more diverged species; supp. fig. 14 and 15).'

Line 258-261: "The 209 samples …… supporting gene conversion occurring within each genome" sounds like the authors are suggesting 209 conversion events. Whereas, they are really invoking only two major events (line 263).

Indeed, to clarify we have updated the text as follows:

'The 209 samples [...]. The strips of near-all beige indicate likely sequence copying between the two genes in a sample, supporting within-genome gene conversion. Two main sets of samples can be distinguished visually, consistent with at least two distinct conversion events (labelled on the right) having occurred in the lineages leading to these samples.'

In doing so, and reading your comments below, we realised that the term 'conversion cluster' was confusing; we realised that really what we wanted to say was that the samples could be divided into two groups, each group showing evidence of the same gene conversion event. We have thus changed 'conversion cluster' to 'conversion event' throughout the paper, including in the right-hand side of Fig. 4 panel b:

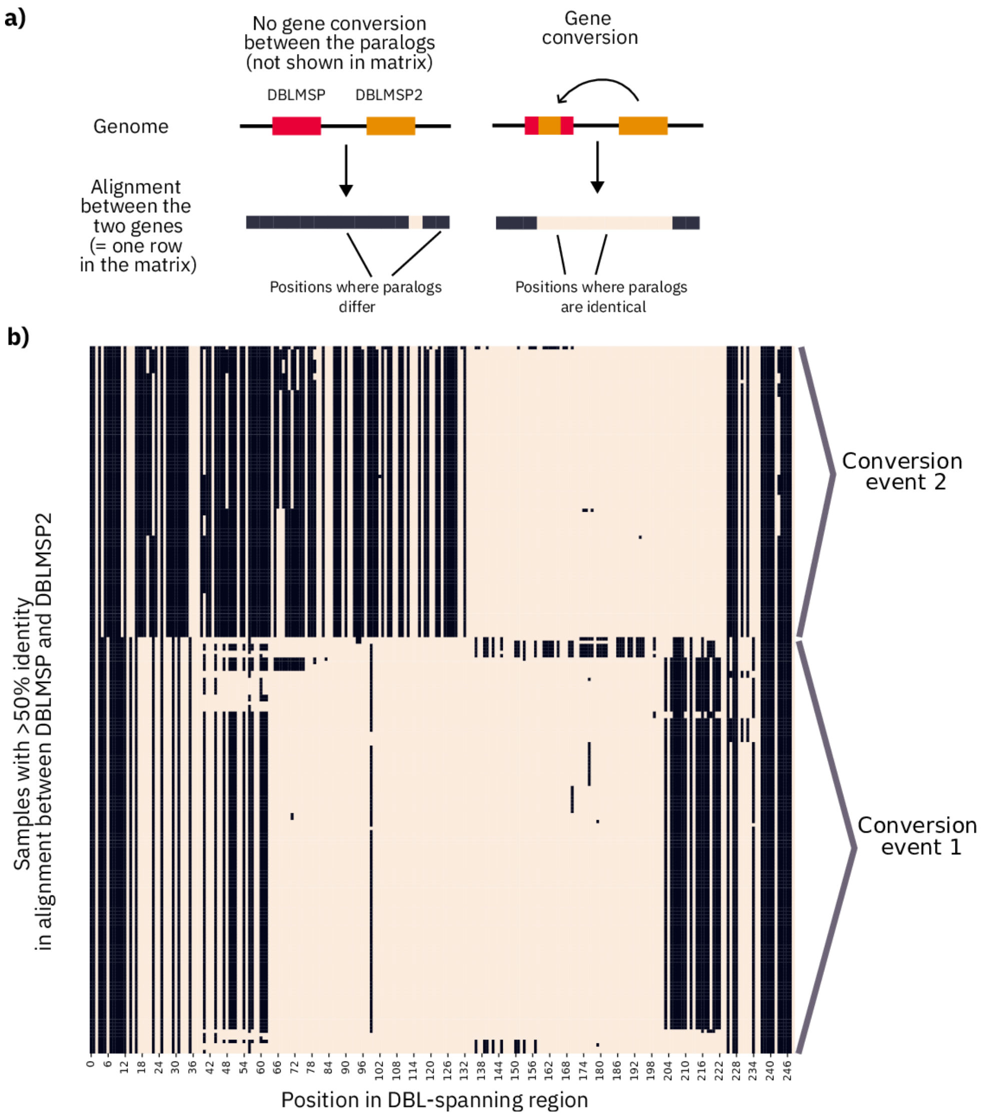

In moving to thinking about events and not clusters and to further clarify the paper, we also decided to modify Fig. 5 panel a in two ways. First, we removed ‘Fraction of identical codons between paralogs’ from Fig. 5 legend, as it was confusing (as per your comment below), and all we want to show in this panel are the two distinct conversion events. We thus labelled samples from the two events in Fig. 4 directly, instead of showing this quantitative ‘fraction’:

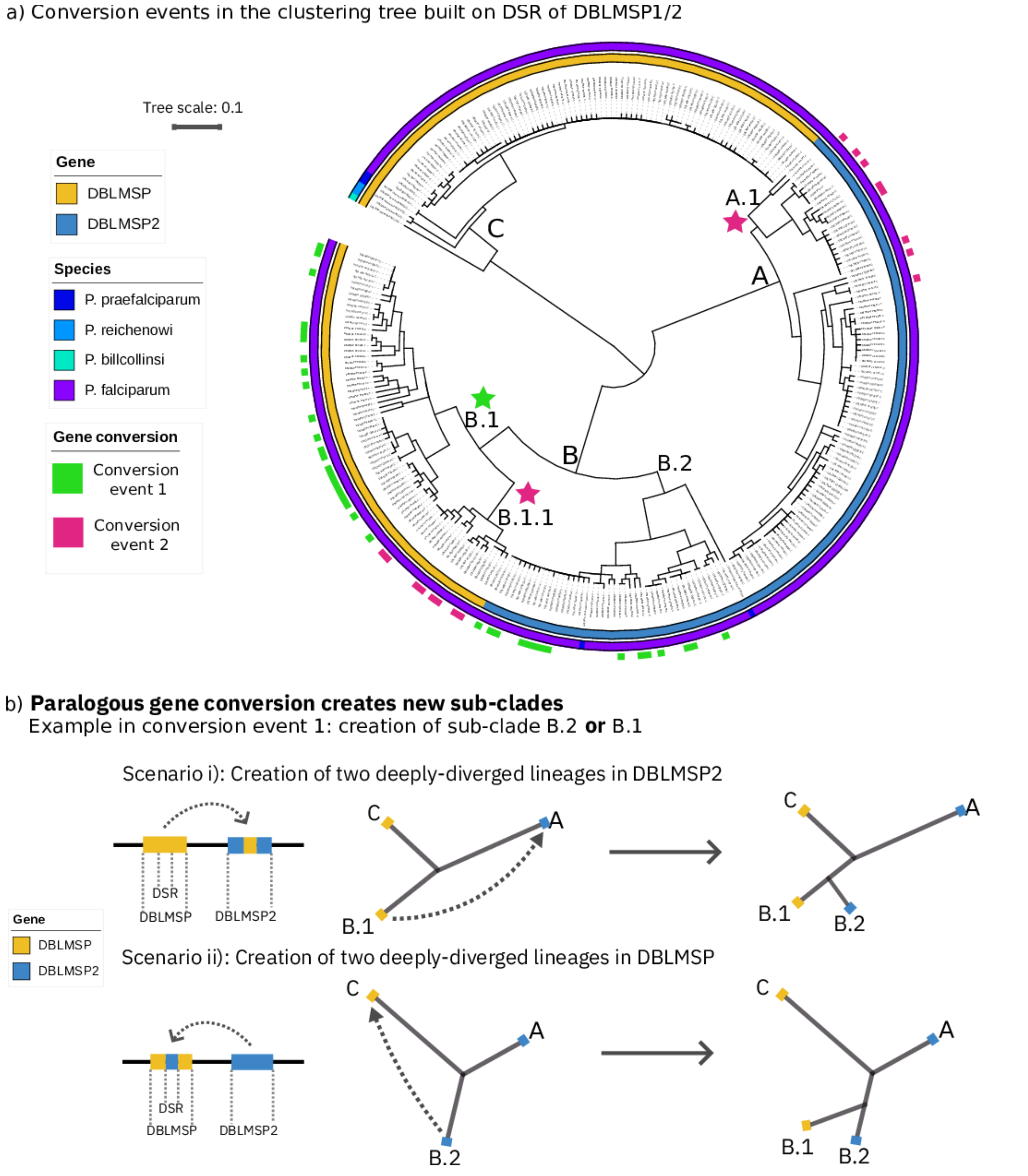

The second modification is that we added coloured stars in panel a) above to indicate which sub-clades the two events gave birth to. Conversion event 1 likely gave rise to sub-clade B.1, and conversion event 2 gave rise to clade A.1 or B.1.1 - we do not know which, as we cannot infer the direction of gene conversion. We find this annotation ties together panels a and b and the main text much better, which now reads as follows:

‘*In Figure 5, we illustrate the relationship between gene conversion and the lineages of DBLMSP1/2. In panel a, we show once more the clustering tree from Figure 2, with the addition of an outer ring marking samples belonging to the two different conversion events identified in Fig. 4. The coloured stars mark the putative locations of new sub-clades in the tree that were created by gene conversion.*

Conversion event 1 is labelled in green in Figure 5 panel a, and panel b illustrates its effect on an ancestral tree of DBLMSP1/2 sequences, depending on whether DBLMSP pasted into DBLMSP2 (scenario i)), or vice-versa (scenario ii). [...]

Similarly, conversion event 2 led to the birth of lineage A.1 or lineage B.1.1, and likely occurred after conversion event 1 as sub-clade B.1.1 lies nested within sub-clade B.1. Here we cannot speculate on which gave rise to the other, so we show two pink stars in Fig. 5 panel a. [...].’

Line 290: I do not understand “we note that paralogous sequences diverge faster than orthologous sequences” ?
In general, at the interspecific level, that would not seem to make sense. Is this comment specific to intraspecific observations, reflecting limited recombination between paralogues?

Thank you for picking up on this, our reasoning was unclear. The goal of the paragraph you referred to was to argue for a specific direction of gene conversion event 1 (namely, from DBLMSP2 to DBLMSP, giving rise to sub-clade B.1). We used to have two rationales for this, and we have decided to just keep the first one, relating to which *P. praefalciparum* alleles exist in the tree in Fig. 5 panel a. We drop the second rationale which you highlight on line 290. Here is the new paragraph in full (highlighted is the retained argument):

‘*Conversion event 1 is labelled in green in Figure 5 panel a, and panel b illustrates its effect on an ancestral tree of DBLMSP1/2 sequences, depending on whether DBLMSP pasted into DBLMSP2 (scenario i)), or vice-versa (scenario ii). For example, in scenario ii), a pre-existing sequence from lineage B.2 in DBLMSP2 pasted into a sequence from DBLMSP lineage C, giving rise to lineage B.1 in DBLMSP and the creation of two deeply-diverged lineages in DBLMSP. In scenario i), two deeply-diverged lineages are instead created in DBLMSP2, through pasting from a DBLMSP allele in lineage B.1. Note that because almost all of the sequence has been pasted (~80%; Fig. 4 panel b), the recipient sequence ends up in a lineage close to the donor sequence; the opposite would hold if a small minority (e.g., 20%) of the sequence had been pasted.* ***We***

***hypothesise scenario ii) is more likely, leading to the birth of sub-clade B.1 (green star in Figure 5 panel a), because we identified sequences from P. praefalciparum in these two lineages (B.2 and C), but no sequences from either lineages B.1 (DBLMSP) or A (DBLMSP2)**.'*

We believe this argument (highlighted in bold) is strong enough on its own (especially given our observation that *P. praefalciparum* alleles cluster with *P. falciparum* alleles as mentioned above, suggesting we have the 'original' *P. falciparum* alleles); and this remains a hypothesis anyway.

Line 296: it is commented that the alleles in conversion cluster 1 do not cluster in the tree, with the excuse that the fraction of pasted sequence is lower. But the alleles in conversion cluster 2 do not cluster in the tree either.

You're right, the alleles of conversion cluster 2 do not cluster but occur close to each other; we have corrected this by using the words 'close to each other' rather than 'cluster': see the sentence in bold in the updated text below. Also, note that (as specified above) we now refer to conversion 'events', not clusters. We have also renumbered, so the old 'conversion cluster 2' is now called 'conversion event 1', as we believe it preceded conversion event 2. This is also stated in the modified main text below:

'*Similarly, conversion event 2 led to the birth of lineage A.1 or lineage B.1.1, and likely occurred after conversion event 1 as sub-clade B.1.1 lies nested within sub-clade B.1. Here we cannot speculate on which gave rise to the other, so we show two pink stars in Fig. 5 panel a.* ***Note that because of the intermediate fraction of pasted sequence for this event, (~0.55; Fig. 4 panel b) sub-clades A.1 and B.1.1 are not located close to each other in the tree.*** *Finally, while our data are clearly consistent with sub-lineage birth in the tree of DBLMSP by gene conversion, they do not explain the pre-existence of deeply-diverged lineages in DBLMSP2 (Fig. 5 panel b). We return to this in the discussion.*'

Figure 5: For clarity: when I saw "Fraction of identical codons between paralogs" I first read this to mean the fraction of codons, within the converted region, that remain identical. Whereas I now believe this to be an indication of the length of the converted region.

Thank you, we have now removed this label entirely; see our response to your comment above, in which we present an updated Figure 5.

Line 378: I think it should be “selected) in”

Yes, thanks and corrected
